# Modelling Stochastic Sensor Noise via Mask-Conditioned Diffusion for Data Augmentation in Low-SNR LGE-CMR

**DOI:** 10.3390/s26102933

**Published:** 2026-05-07

**Authors:** Sofia Fernandes, Carla Barros, Adriano Pinto, Vitor H. Pereira, Carlos Lima, Carlos A. Silva

**Affiliations:** 1 Department of Industrial Electronics, School of Engineering, University of Minho, Campus de Azurém, 4800-058 Guimarães, Portugal; carlos.lima@dei.uminho.pt; 2 CMEMS-UMinho, Department of Industrial Electronics, School of Engineering, University of Minho, Campus de Azurém, 4800-058 Guimarães, Portugal; 3 AI4MedImaging Medical Solutions, 4705-002 Braga, Portugalvpereira@ai4cmr.com (V.H.P.); 4 Life and Health Sciences Research Institute (ICVS), School of Medicine, University of Minho, Campus de Gualtar, 4710-057 Braga, Portugal; 5 LABBELS—Associate Laboratory, Braga, 4710-057 Guimarães, Portugal

**Keywords:** LGE-CMR, stochastic noise modelling, diffusion models, data augmentation, signal reconstruction, myocardial fibrosis

## Abstract

Late gadolinium enhancement cardiovascular magnetic resonance (LGE-CMR) permits non-invasive quantification of myocardial fibrosis; however, automated scar segmentation remains challenging due to limited expert annotations and reduced image quality caused by acquisition noise and artefacts. We investigate two related questions: (i) whether inversion of a stochastic Gaussian diffusion process can reproduce the texture characteristics of low-signal-to-noise-ratio (SNR) LGE imaging, and (ii) whether the resulting synthetic data can improve automated fibrosis segmentation in annotation-limited settings. To this end, we introduce a mask-conditioned denoising diffusion probabilistic model (DDPM) that synthesises high-fidelity 2D short-axis LGE-CMR slices from three-class label maps (background, myocardium, scar), and we employ these synthetic images for training-set augmentation. The impact of augmentation was assessed using the nnU-Net v2 segmentation framework and benchmarked against exemplar-guided image synthesis with CoCosNet-v2 under identical data partitioning. On a held-out test set trained with 100 real cases, inclusion of 300 diffusion-generated cases increased the scar Dice coefficient from 0.173 to 0.271 (+56.7%), and the scar recall from 0.173 to 0.363, demonstrating enhanced sensitivity to fibrotic lesions. For comparable training budgets, diffusion-based augmentation consistently outperformed GAN-based augmentation, although performance improvements were non-monotonic with respect to the real-to-synthetic data ratio and attenuated as the size of the real dataset increased. A four-axis noise-fidelity analysis (spectral content, signal-dependent variance, short-range spatial correlation, distributional shape) further shows that the DDPM reproduces scanner-specific noise statistics substantially more faithfully than the GAN baseline, providing a mechanistic account for the augmentation gap.

## 1. Introduction

Cardiovascular diseases represent a leading cause of mortality worldwide, highlighting the necessity for ongoing advances in diagnostic imaging [1]. Cardiac magnetic resonance (CMR) is widely regarded as the reference standard for comprehensive cardiac assessment, functioning as a high-fidelity imaging sensor that enables both qualitative and quantitative characterisation of myocardial pathology. Late gadolinium enhancement (LGE) provides high-resolution assessment of myocardial structural integrity and facilitates the detection of ischaemic heart disease and cardiomyopathies through the identification of fibrotic tissue [1]. The LGE contrast mechanism, which exploits the differential tissue retention of gadolinium-based contrast agents, augments signal contrast in fibrotic myocardium, thereby rendering these regions hyperintense in the reconstructed images [2].

Despite its clinical importance, accurate segmentation of myocardial structures and fibrotic regions in LGE-CMR remains a non-trivial task. Fibrosis often exhibits heterogeneous spatial distributions, and stochastic noise, artefacts, and variability in sensor acquisition protocols further complicate robust automation. Manual delineation is still the prevalent practice, but it is time-consuming and susceptible to inter- and intra-observer variability. These challenges are exacerbated by the limited availability of expert-annotated datasets [1], as the generation of pixel-wise labels demands specialised expertise and substantial annotation time.

Deep learning-based methods have demonstrated high performance in medical image analysis; however, their generalisation capability is strongly dependent on the volume and quality of training data. While pooling data across multiple institutions could enhance cohort heterogeneity, legal, ethical, and privacy constraints often restrict the sharing of medical data. A promising alternative is the enrichment of existing datasets through synthetic data generation [3]. Recent progress in generative modelling, particularly in deep learning-based approaches, has established these methods as powerful tools for addressing several long-standing challenges in medical imaging: (i) the limited availability of expert-annotated datasets arising from costly data acquisition and labour-intensive labelling processes; (ii) class imbalance resulting from the low prevalence of many pathologies; (iii) privacy and data-governance constraints that impede large-scale data sharing; (iv) heterogeneity and distribution shifts across imaging devices, acquisition protocols, and patient populations that hinder model generalisation; and (v) imperfect measurements (e.g., noise, artefacts, and undersampling) that necessitate denoising and inverse-problem formulations [3]. Previous studies have employed generative adversarial networks (GANs) for medical image synthesis; nevertheless, GAN training is frequently unstable and may not adequately capture the underlying probability distribution of the imaging data, often leading to mode collapse [3].

Denoising diffusion probabilistic models (DDPMs) learn a stochastic denoising process that progressively transforms random noise into high-fidelity images. Compared with GANs, diffusion models are typically associated with improved sample diversity and offer flexible conditioning strategies for controlled image synthesis, properties that are particularly advantageous in data-constrained scenarios [4]. Furthermore, recent findings in the field of medical image synthesis demonstrate that DDPMs are capable of producing high-quality outputs while preserving fine-grained images, frequently exhibiting fewer reconstruction artefacts and superior fidelity compared to GAN-based methods [5].

Motivated by these considerations, the present study investigates mask-conditioned diffusion models for synthetic LGE-CMR generation as a data augmentation strategy for myocardial fibrosis segmentation. We perform a quantitative assessment of the effect of diffusion-based augmentation on the robustness and accuracy of myocardium and scar segmentation in a limited-annotation regime, and benchmark it against an exemplar-guided GAN baseline under identical downstream training conditions.

### 1.1. Myocardial Fibrosis Segmentation in LGE-CMR

Automated segmentation of myocardial fibrosis and related pathological substrates, namely myocardial infarction (MI) and microvascular obstruction (MVO), in LGE-CMR is of high clinical relevance, as both the presence and extent of scar tissue are strongly associated with adverse outcomes, including left-ventricular (LV) dysfunction and ventricular arrhythmogenesis [6]. However, robust delineation of these substrates remains technically challenging due to (i) substantial overlap in intensity distributions between pathological tissue and adjacent anatomical structures, most notably the hyper-enhanced blood pool; (ii) marked class imbalance between healthy myocardium and pathological regions; and (iii) pronounced inter-subject and inter-acquisition variability, which is further aggravated by motion artefacts and atypical ventricular geometries [7,8,9]. As a result, the availability of generalisable, fully automated pipelines for routine two-dimensional LGE analysis remains limited, primarily because of the paucity of large, heterogeneous, and rigorously annotated datasets [9].

From a methodological standpoint, recent literature can be broadly categorised into several principal directions: (a) classical and hybrid approaches that integrate image registration and atlas-based initialisation with geometric constraints and probabilistic intensity models [10,11,12,13]; (b) convolutional neural network (CNN) and U-Net derivatives incorporating attention mechanisms, task-specific loss functions, and self-configuring training frameworks such as nnU-Net [14,15,16]; (c) cascaded or region-of-interest (ROI)-constrained architectures that exploit anatomical priors and inclusion constraints (MVO ⊂ MI ⊂ myocardium), often combined with error-tolerant refinement stages [7,17,18,19,20]; (d) multi-sequence or multimodal learning strategies that integrate cine, T2-weighted, or balanced steady-state free precession (bSSFP) imaging to mitigate contrast ambiguities inherent to LGE alone [21,22,23,24]; (e) domain adaptation and harmonisation techniques, including approaches targeting robustness to variations in acquisition protocols and spatial resolution [25,26,27]; and (f) semi-supervised learning frameworks based on consistency regularisation and teacher–student paradigms to leverage partially labelled data [28].

### 1.2. Diffusion Models for Data Augmentation and Segmentation

Generative data augmentation has been extensively explored as a methodology to mitigate challenges arising from limited annotation availability and distributional shifts across imaging centres. Earlier studies in LGE-CMR primarily employed GANs for segmentation-conditioned image synthesis and style transfer [29,30]. Nevertheless, GAN-based approaches frequently exhibit difficulties in adequately modelling the full tail of the underlying data distribution, which is particularly consequential for reliably representing rare pathological phenotypes.

More recently, DMs have gained substantial prominence due to their capacity for high-fidelity sample synthesis, improved generative diversity, and theoretically well-founded mechanisms for controllable generation via conditioning [3,31,32]. From a signal processing standpoint, DMs conceptualise data generation as a reverse-time diffusion process, in which the model learns to reconstruct the underlying image signal from Gaussian noise. In practice, many pipelines employ latent diffusion or slice-wise formulations to mitigate computational complexity, and the usefulness of the synthesised images is typically evaluated in terms of their effect on downstream segmentation performance [33,34,35].

In parallel, diffusion-based approaches have been explored as segmentation frameworks by recasting mask prediction as an iterative denoising inference process. This approach allows the model to learn complex spatial correlations even in settings with limited labelled data [30,36].

The available evidence for diffusion-based data augmentation specifically adapted to standard 2D LGE-CMR remains sparse, particularly in the presence of pronounced class imbalance and substantial protocol heterogeneity, both of which exacerbate the need for methodological robustness. In this study, we systematically evaluate diffusion models for the synthetic reconstruction of LGE signals encompassing both normal and pathological cases. We quantitatively assess the effect of diffusion-driven augmentation on the robustness and accuracy of automated myocardium and scar segmentation under conditions of limited manual annotation.

## 2. Materials and Methods

### 2.1. Overview

We investigate diffusion-based synthetic LGE-CMR generation as a data augmentation strategy for myocardial fibrosis segmentation. The proposed framework consists of: (i) a mask-conditioned diffusion generator trained on 2D short-axis slices; (ii) an exemplar-guided image-to-image translation baseline (CoCosNet-v2 [37]) for comparative analysis; and (iii) a controlled segmentation study using nnU-Net v2 [38], in which only the composition of the training set is modified, while the validation and test partitions remain fixed.

### 2.2. Datasets

The LGE-CMR dataset used in this study comprises imaging data from 1509 patients (63% male, 37% female), corresponding to approximately 14,000 short-axis slices. Only magnitude images were utilised in all experimental procedures. Patients were retrospectively included if their examination contained short-axis slices, ensuring adequate coverage of the left ventricle. All examinations were acquired in a routine clinical workflow at a tertiary care centre and represent patients referred for CMR owing to suspected cardiac disease. As a result, the dataset encompasses a heterogeneous and clinically representative population, including cases with normal findings, as well as ischaemic and non-ischaemic myocardial pathology. All examinations were performed on Siemens magnetic resonance imaging systems operating at field strengths of 1.5 T and 3 T, utilising a standardised late gadolinium enhancement cardiovascular magnetic resonance (LGE-CMR) protocol based on inversion-recovery imaging techniques.

In-plane spatial resolution was largely homogeneous across examinations: 84% of cases had a pixel spacing of 1.77×1.77 mm, 15% had 1.82×1.82 mm, and 1% had values ranging from 1.56×1.56 mm to 1.68×1.68 mm. The image matrix size varied accordingly, with the majority of images measuring 192×176 pixels. Slice thickness was 8 mm and inter-slice spacing 10 mm for the majority of examinations.

To establish reference standards for myocardial segmentation and fibrosis detection, all images were manually annotated using a dedicated, custom-developed annotation software. The labelling process, including delineation of endocardial and epicardial contours as well as identification of regions of gadolinium enhancement, was performed by an expert in CMR. The dataset consists of anonymised routine clinical imaging data owned by the authors’ institution, and all relevant ethical, privacy, and data protection regulations were satisfied prior to its use in this study.

From the complete cohort, a subset of 600 patients (approximately 5405 short-axis slices) was selected for development of the DDPM. Myocardial fibrosis consistent with LGE was present in approximately 55% of these patients (i.e., detectable in at least one slice), with cases exhibiting non-ischaemic and ischaemic fibrosis patterns. All images had an in-plane pixel spacing of 1.77 × 1.77 mm.

For nnU-Net training, we included 1230 patients from the full cohort, among whom myocardial fibrosis was present in approximately 17%. To avoid data leakage, we implemented patient-level separation between the DDPM training set and the nnU-Net test set, ensuring that no patient contributing to diffusion model training was included in the segmentation evaluation cohort.

### 2.3. Data Pipeline and Pre-Processing

Training pairs $\left(x_{0},c\right)$ are extracted on a per-slice basis from the volumetric NIfTI datasets using a custom data loader that identifies and selects clinically relevant short-axis slices together with their corresponding annotations. Image intensities are initially normalised via min–max scaling to the interval [0,1] and subsequently linearly rescaled to [−1,1], while segmentation masks are transformed into one-hot encoded, multi-channel representations. Finally, a deterministic ROI-based cropping procedure is applied to each image–mask pair in order to obtain inputs of image size 128×128 pixels.

### 2.4. Mask-Conditioned Diffusion Synthesis

#### 2.4.1. DDPM Formulation

**Figure 1:**
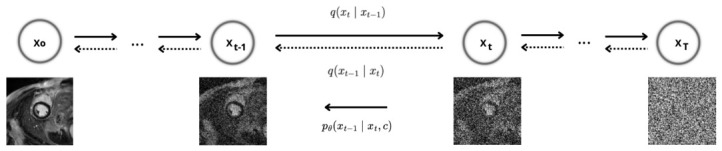
Overview of a DDPM. The arrows indicate the direction of the forward noising and reverse denoising processes. The forward process progressively corrupts a clean LGE slice $x_{0}$ into $x_{T}$ by adding Gaussian noise. The learned reverse process iteratively denoises the image, sampling $x_{t-1}$ from $p_{\theta }\left(x_{t-1}\;|x_{t},c\right)$ conditioned on the segmentation mask *c*, until obtaining an estimate of $x_{0}$.

A mask-conditioned DDPM is employed for LGE synthesis. Figure 1 provides an overview of both the forward noising process and the reverse denoising process. In our framework, the reverse-time dynamics are explicitly conditioned on the segmentation mask *c*.

DDPMs define a forward Markovian diffusion process that progressively perturbs a clean image signal by the addition of Gaussian noise, and they learn the corresponding reverse-time dynamics for signal reconstruction [6,39,40]. Let $x_{0}\in R^{1\times H\times W}$ denote an LGE slice sampled from the empirical data distribution. The forward diffusion process, modelled as a series of noise-injection steps, is given by

(1) $$q\left(x_{t}|x_{t-1}\right)=N\;\left(x_{t};\sqrt{1-\beta_{t}}\;x_{t-1},\;\beta_{t}I\right),\;t=1,\ldots ,T,$$

where ${\left\{\beta_{t}\right\}}_{t=1}^{T}$ is a predefined noise-variance schedule, $\alpha_{t}=1-\beta_{t}$, and ${\bar{\alpha }}_{t}=\prod_{i=1}^{t}\alpha_{i}$. This construction admits the following closed-form expression for the marginal distribution at time step *t*:

(2) $$q\left(x_{t}|x_{0}\right)=N\;\left(x_{t};\sqrt{{\bar{\alpha }}_{t}}\;x_{0},\;\left(1-{\bar{\alpha }}_{t}\right)I\right),$$

which enables direct sampling of noisy realisations at arbitrary timesteps during training via

(3) $$x_{t}=\sqrt{{\bar{\alpha }}_{t}}\;x_{0}+\sqrt{1-{\bar{\alpha }}_{t}}\;\varepsilon ,\;\varepsilon ∼N\left(0,I\right).$$

In our implementation, timesteps are indexed in a zero-based manner, i.e., t∈{0,…,T−1}, whereas the formulation above follows the conventional t=1,…,T notation.

#### 2.4.2. Mask Conditioning

We employ a conditional diffusion model based on Med-DDPM [41], which we adapt to a slice-wise setting operating on short-axis LGE-CMR images. Let $c\in {\left\{0,1\right\}}^{3\times H\times W}$ denote the corresponding one-hot encoded segmentation mask with three classes (background, myocardium, scar). The conditioning is realised by channel-wise concatenation of the image and the segmentation mask at each diffusion step:

(4) $${\tilde{x}}_{t}=\mathrm{concat}\left(x_{t},c\right).$$

#### 2.4.3. Reverse Process

At each timestep, t ∈ {0, …, T−1}, the denoising network predicts the noise component from the conditioned input ${\tilde{x}}_{t}$. The reverse diffusion process is modelled as a Gaussian transition of the form

(5) $$p_{\theta }\left(x_{t-1}|x_{t},c\right)=N\;\left(x_{t-1};\;\mu_{\theta }\left(x_{t},t,c\right),\;\sigma_{t}^{2}I\right),$$

where the denoising network estimates the noise term $\varepsilon_{\theta }\left({\tilde{x}}_{t},t\right)$ from the conditioned input ${\tilde{x}}_{t}$.

Adopting the ε-parameterisation commonly used in DDPMs, the reverse-process mean can be expressed as

(6) $$\mu_{\theta }\left(x_{t},t,c\right)=\frac{1}{\sqrt{\alpha_{t}}}\left(x_{t}-\frac{1-\alpha_{t}}{\sqrt{1-{\bar{\alpha }}_{t}}}\;\varepsilon_{\theta }\left({\tilde{x}}_{t},t\right)\right),$$

and the corresponding sampling step is given by

(7) $$x_{t-1}=\mu_{\theta }\left(x_{t},t,c\right)+\sqrt{\beta_{t}}\;z,\;z∼N\left(0,I\right),$$

such that no additional stochastic perturbation is introduced at t = 0 [39,40].

#### 2.4.4. Training Objective and Architecture

We employ a cosine β-schedule with T=250 diffusion steps and optimise the model under the standard noise-prediction objective. At each iteration, we sample a diffusion timestep t∼U{0,…,T−1} and construct the noised sample

$$x_{t}=\sqrt{{\bar{\alpha }}_{t}}\;x_{0}+\sqrt{1-{\bar{\alpha }}_{t}}\;\varepsilon ,\;\varepsilon ∼N\left(0,I\right).$$

The denoising network is trained to minimise the objective

(8) $$L_{\mathrm{simple}}=E_{x_{0},c,t,\varepsilon }\left[\ell \;\left(\varepsilon ,\varepsilon_{\theta }\left(\mathrm{concat}\left(x_{t},c\right),t\right)\right)\right],$$

where $\ell (\varepsilon ,\hat{\varepsilon })$ denotes either the per-pixel $\ell_{1}$ distance or the mean squared error ($\ell_{2}$). In our experiments, we adopt the $\ell_{1}$ variant, following reports from the original authors indicating that this choice yields superior empirical performance.

Although raw MRI data theoretically follow a Rician noise distribution, at the signal-to-noise ratio (SNR) levels typically observed in myocardial tissue on reconstructed magnitude images, the scanner’s noise distribution can be approximated as a moment-matched Gaussian distribution. We verify this approximation empirically for the present cohort in Section 3.3. The reverse process of the diffusion model learns to predict the residual noise, thereby capturing the texture and noise grain characteristic of LGE acquisitions. Furthermore, conditioning on the segmentation mask constrains the model to learn the noise texture within the ROI, rather than merely the anatomical shape of the underlying structures.

### 2.5. Diffusion Model

**Figure 2:**
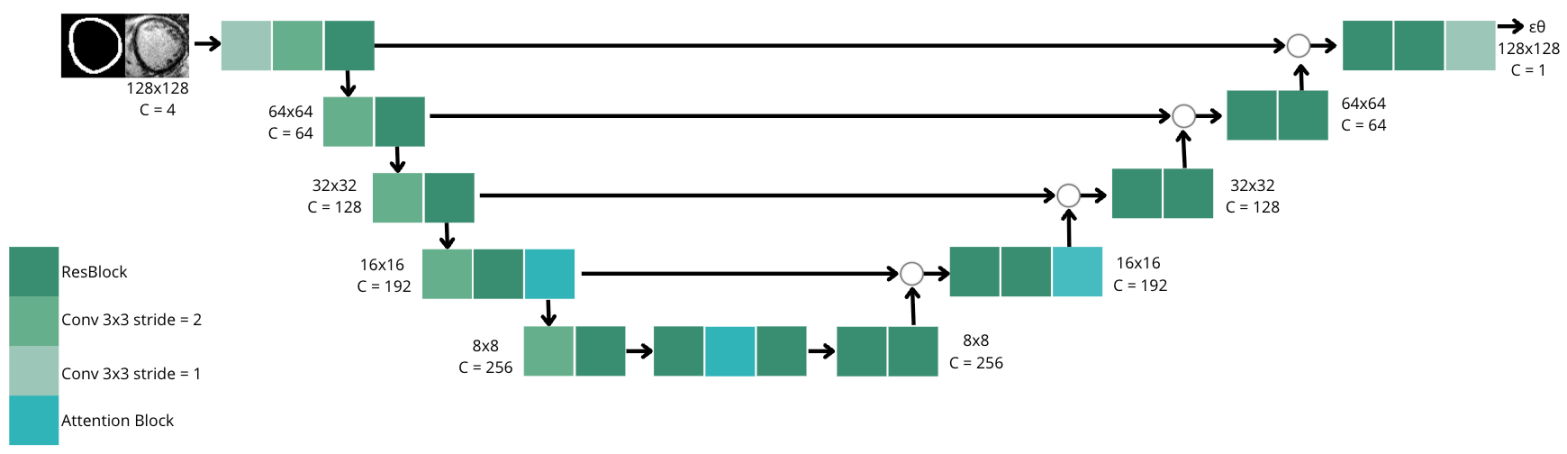
Architecture of the 2D U-Net denoiser used in the mask-conditioned DDPM. Arrows indicate feature propagation through the encoder, bottleneck, decoder, and skip connections. Coloured blocks denote residual, convolutional, and attention modules, while circles indicate skip-connection fusion points. The output corresponds to the predicted noise.

The denoising component is instantiated as a two-dimensional U-Net architecture incorporating residual blocks across multiple resolution scales, sinusoidal timestep embeddings, and attention mechanisms at coarser spatial resolutions to model long-range spatial dependencies effectively, as shown in Figure 2. Model training is conducted with a base channel width of 64 and one residual block per resolution level.

#### 2.5.1. Imbalance-Aware Objective

To mitigate class imbalance and enhance the small pathological features, we employ a spatially weighted loss function whenever corresponding segmentation annotations are available. Specifically, a pixel-wise weight map *W* is constructed from the one-hot encoded segmentation mask *Y*:

(9) $$W\left(i,j\right)=\sum_{c\in \{\mathrm{bg},\;\mathrm{myo},\;\mathrm{scar}\}}w_{c}\;Y_{c}\left(i,j\right),$$

where W(i,j) denotes the weight assigned to pixel (i,j), *c* indexes the semantic classes (background, myocardium, scar), $w_{c}\in R_{+}$ is the non-negative scalar weight associated with class *c*, and $Y_{c}\left(i,j\right)\in \left\{0,1\right\}$ is the corresponding binary class indicator (i.e., $Y_{c}\left(i,j\right)=1$ if pixel (i,j) belongs to class *c*, and 0 otherwise).

The class weights $w_{c}$ are determined empirically through hyperparameter optimisation on a held-out validation set, and the final values are documented in the Appendix A. As an illustrative example, one configuration employs $w_{\mathrm{bg}}=0.2$, $w_{\mathrm{myo}}=1.5$, and $w_{\mathrm{scar}}=3.0$.

The training objective is then defined as a normalised, spatially weighted average:

$$L=\frac{\sum_{i}w_{i}\;\ell_{i}}{\sum_{i}w_{i}+\epsilon },$$

where $\ell_{i}$ is the per-pixel noise-prediction loss and ϵ is a small constant introduced to ensure numerical stability.

#### 2.5.2. Optimisation and Checkpointing

Training was performed for 150,000 optimisation iterations. Checkpoints and intermediate samples were produced every 1000 iterations. An exponential moving average (EMA) of the model parameters with decay rate 0.995 was maintained and used for sampling. At each sampling milestone, 50 images were generated and saved, and the Fréchet inception distance (FID) [42] was computed between the generated samples directory and a fixed reference directory. The checkpoint achieving the lowest FID was retained as the best-performing generator.

#### 2.5.3. Implementation Details

Optimisation was performed using the Adam algorithm with a fixed learning rate of $1\times 10^{-5}$, without learning-rate scheduling or weight decay. A mini-batch size of 4 was used with gradient accumulation over 2 iterations, resulting in an effective batch size of 8. The diffusion process was parameterised with T=250 discrete timesteps, and all input images were isotropically rescaled to a spatial resolution of 128×128 pixels. The denoising U-Net architecture was instantiated with a base feature dimensionality of 64 channels and a single residual block at each resolution level. During training, an exponential moving average (EMA) of the network parameters with a decay coefficient of 0.995 was maintained and subsequently used for model evaluation and sample generation (Additional implementation and computational details are provided in Appendix A).

##### Determinism and Early Stopping

Reproducibility is facilitated by enforcing deterministic training conditions, including the use of fixed random seeds and deterministic data augmentation procedures. Furthermore, early stopping is implemented by monitoring the exponentially smoothed validation pseudo-Dice metric, with a patience threshold set to 100 epochs.

### 2.6. Benchmark: Exemplar-Guided Synthesis (CoCosNet-v2)

To complement the diffusion-based generative models, we employ CoCosNet-v2 [37] as an exemplar-guided image-to-image translation baseline, used exclusively for comparative benchmarking. All experiments are performed in a two-dimensional setting on myocardium-centred image patches of spatial resolution 128×128. The dataset consists of (image, mask) pairs, where the segmentation mask is one-hot encoded into three classes (background, myocardium, and scar). To preserve compatibility with the original CoCosNet-v2 feature extraction layers, each single-channel image slice is replicated across three channels prior to being input to the generator network.

#### 2.6.1. Reference Matching

To reduce anatomical discrepancies between target and exemplar slices, we define two reference pools: normal and, optionally, scar. Pool membership is determined by the spectral properties of the target mask: a slice is assigned to the scar pool if the scar channel (label 2) contains at least one positive voxel, and to the normal pool otherwise. In the absence of scar references, scar-containing target slices are matched against the normal pool.

Reference slices are not sampled uniformly at random; instead, we perform slice-level matching based on myocardial size. For each slice, the myocardial ROI is derived from the myocardium. The exemplar slice is then selected using a nearest-neighbour criterion that minimises the absolute difference between the myocardial area of the target slice and that of candidate reference slices within the corresponding pool, thereby reducing geometric noise in the translation process.

#### 2.6.2. Training and Model Selection

To ensure strict comparability with diffusion-based model selection, CoCosNet-v2 training is monitored using an explicit FID-based protocol. A fixed subset of K=50 target samples is selected once using a deterministic random seed, and the corresponding real target images are exported to constitute a persistent reference set. At the end of each training epoch, the model synthesises images for this fixed subset, and computes FID with respect to the fixed real-reference. During training, we periodically evaluate the model using the FID and designate the checkpoint achieving the optimal (i.e., lowest) FID value as the final generator. Training is performed with a batch size of 4, a learning rate of $10^{-4}$.

### 2.7. Segmentation Evaluation (nnU-Net)

For downstream fibrosis segmentation, we adopt nnU-Net [38] as the reference segmentation framework. nnU-Net is employed exclusively in its 2D configuration, and all experiments are performed with a fixed batch size of 35 to ensure computational consistency across training conditions. To standardise the field of view and constrain model capacity to clinically relevant anatomy, all inputs are cropped to a fixed 64×64 myocardium-centred ROI.

#### Controlled Augmentation Protocol

The impact of synthetic augmentation is investigated under a controlled experimental protocol. The validation and test subsets are held fixed to provide a consistent evaluation benchmark, while the training-set composition (real versus synthetic data) is varied. Segmentation performance is quantified using Dice coefficient, precision, and recall, with particular emphasis on the scar class due to its low signal prevalence and morphological complexity.

### 2.8. Experimental Setup

All results are obtained under a fixed evaluation protocol with a held-out validation set (n=120 patients) and test set (n=120 patients). For each training-set composition, we run a single deterministic nnU-Net training to isolate the effect of dataset composition and ensure direct comparability across conditions. A detailed overview of the patient-level dataset partitioning strategy employed for diffusion model development and subsequent segmentation experiments is presented in the Table A1.

### 2.9. Evaluation Metrics

We assess (i) the fidelity of the synthesised images and (ii) the performance on the downstream segmentation task. For the evaluation of generative quality, we report Fréchet inception distance (FID), kernel inception distance (KID), structural similarity index measure (SSIM), peak signal-to-noise ratio (PSNR), and learned perceptual image patch similarity (LPIPS). In addition, we include the inception score (IS) as a complementary metric to characterise sample quality and diversity.

FID was computed using the TorchMetrics implementation, employing the default Inception-v3 network as the feature extractor with a 2048-dimensional embedding space. Both real and generated images were first converted to three-channel inputs, normalised to the [0,1] range, and internally resized according to the input specifications of the Inception network before estimating the feature-space statistics. KID was computed within the same feature space and serves as a kernel-based metric that constitutes an alternative to FID.

SSIM and PSNR were employed to quantify structural fidelity and similarity at the intensity level, respectively, whereas LPIPS was utilised as a perceptual similarity measure. For FID, KID, and LPIPS, lower scores indicate a closer correspondence between generated and reference images, whereas for SSIM, IS, and PSNR, higher values are indicative of superior image generation performance.

Downstream segmentation performance is quantified using the Dice similarity coefficient (DSC), precision, recall, and the 95th-percentile Hausdorff distance (HD95) for both myocardium and scar classes. DSC, precision, and recall characterise spatial overlap, false-positive rate, and sensitivity, respectively, whereas HD95 constitutes a boundary-aware measure of segmentation error by capturing the 95th percentile of the bidirectional distances between the predicted and reference contours. Lower HD95 values correspond to improved spatial congruence between delineated boundaries. DSC, precision, and recall are computed from voxel-wise or pixel-wise counts of true positives (TPs), false positives (FPs), and false negatives (FNs):

(10) $$\mathrm{Dice}=\frac{2\;\mathrm{TP}}{2\;\mathrm{TP}+\mathrm{FP}+\mathrm{FN}},$$

(11) $$\mathrm{Precision}=\frac{\mathrm{TP}}{\mathrm{TP}+\mathrm{FP}},\;\mathrm{Recall}=\frac{\mathrm{TP}}{\mathrm{TP}+\mathrm{FN}}.$$

The classical Hausdorff distance is defined as

(12) H(A,B)=max(h(A,B),h(B,A)),

where

(13) $$h\left(A,B\right)=\max_{a\in A}\min_{b\in B}∥a-b∥.$$

In this work, we use its robust variant, the 95th-percentile Hausdorff distance (HD95), defined as

(14) $$\mathrm{HD}95\left(A,B\right)=\max\left({\mathrm{percentile}}_{95}\left(\{\min_{b\in B}∥a-b∥:a\in A\}\right),{\mathrm{percentile}}_{95}\left(\{\min_{a\in A}∥b-a∥:b\in B\}\right)\right).$$

### 2.10. Statistical Analysis

All statistical analyses were conducted on per-patient performance metrics computed on the fixed held-out test set. Myocardium and scar were analysed separately, given their marked differences in anatomical size, prevalence, and segmentation complexity. The DSC was designated as the primary outcome metric, whereas the 95th-percentile Hausdorff distance (HD95) served as the principal boundary-aware secondary metric.

To quantify uncertainty in the aggregate performance estimates, 95% bootstrap confidence intervals (CIs) were derived for all reported metrics and experimental conditions using 10,000 resamples with replacement from the per-patient values.

Pairwise differences in segmentation performance were assessed using the Wilcoxon signed-rank test (two-sided), applied to paired per-patient metric values. This non-parametric test was chosen, given the skewed distribution of scar Dice scores and the small number of test cases. The test was applied to the following comparisons: (i) Each reduced real-data condition (100, 300, 600 real cases) vs. the full-data baseline (1230 real cases), independently for myocardium and scar. (ii) Three-way focused comparison: no augmentation vs. Diffusion augmentation vs. GAN augmentation. (iii) Loss-weighting ablation in the low-data diffusion regime: weighted vs. unweighted diffusion training loss, evaluated for each synthetic-data increment in the $N_{\mathrm{real}}=100$ setting.

For the three-way comparison, *p*-values were corrected for multiple comparisons using the Holm–Bonferroni method, applied to the three pairwise tests within each tissue independently. The corrected significance threshold was α=0.05. For the reduced real vs. baseline analysis, each comparison constitutes a distinct experimental question, and no additional correction was applied.

For the loss-weighting ablation, paired comparisons between the weighted and unweighted diffusion-loss variants were performed using the Wilcoxon signed-rank test on per-patient segmentation metrics. This analysis was restricted to the low-data regime ($N_{\mathrm{real}}=100$), where class imbalance is expected to have the largest impact. Holm–Bonferroni correction was applied across the five synthetic-data increments within each tissue and metric combination. The same corrected significance threshold (α=0.05) was used. Results from this ablation are reported in the Appendix D, Table A7.

Effect size was quantified using the rank-biserial correlation (*r*), computed as r=1−(2W)/(n(n+1)), where *W* is the Wilcoxon test statistic and *n* is the number of paired observations. Values of |r|≥0.1, 0.3, and 0.5 were considered small, moderate, and large effects, respectively.

To evaluate sensitivity to random initialisation, additional repeated-seed experiments were performed for diffusion-based augmentation in the low-data regime ($N_{\mathrm{real}}=100$). Five independent runs were conducted for each evaluated synthetic-data condition, and results were summarised as mean ± standard deviation and coefficient of variation, as reported in the Section 3.5.2.

## 3. Results and Discussion

### 3.1. Qualitative Evaluation of Mask-Conditioned Synthesis

Before presenting the quantitative segmentation results, we first conduct a qualitative evaluation of representative images synthesised by the proposed mask-conditioned diffusion model. At inference time, the model is initialised with Gaussian noise and conditioned solely on the segmentation mask *c*, thereby generating an LGE-CMR slice in the absence of any paired reference image. For comparison, we also show the corresponding image produced by the exemplar-guided GAN under the same mask-conditioning configuration.

**Figure 3:**
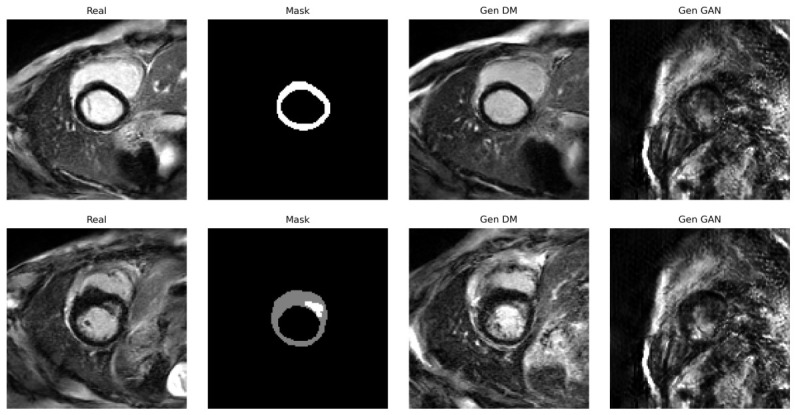
The first row illustrates a case without evidence of fibrotic scarring, whereas the second row depicts a case characterised by the presence of fibrotic scar tissue.

**Figure A1:**
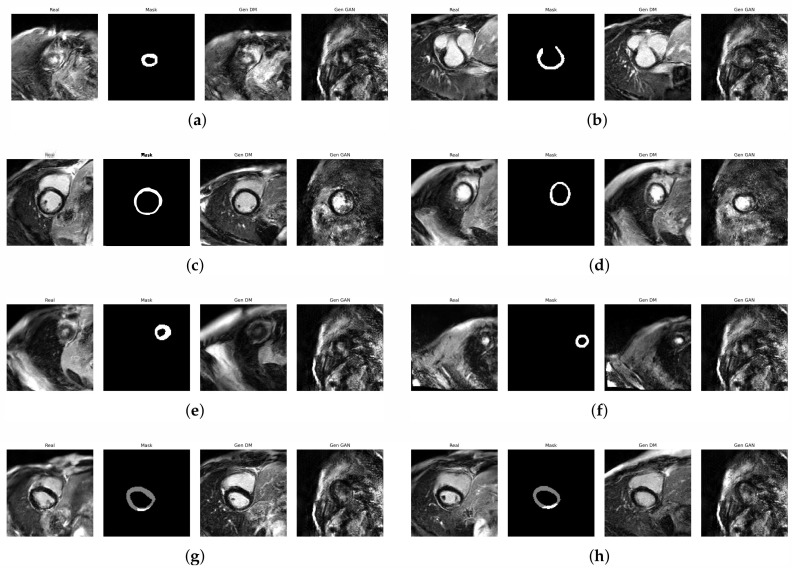

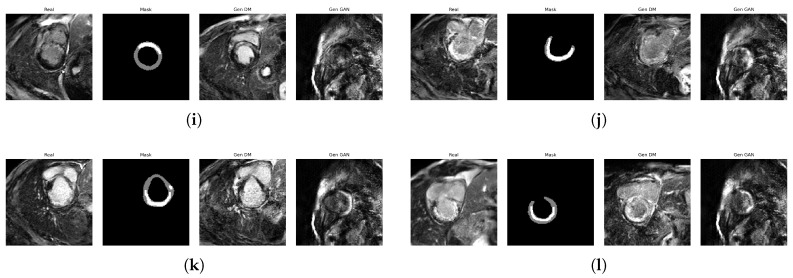
Additional qualitative examples of generated images from multiple subjects and slice locations. In each panel, the images are arranged as Real | Mask | DM | GAN. Panels (**a**–**f**) show cases without visually appreciable scar tissue, whereas panels (**g**–**l**) show cases with visible scar tissue. These examples illustrate the real image, the corresponding conditioning mask, and the images synthesised by the diffusion-based and GAN-based generators under both non-scar and scar-present conditions.

Figure 3 depicts two representative examples. The first row corresponds to a non-pathological case without fibrotic scar, characterised by a myocardium-only mask, whereas the second row corresponds to a pathological case with visible fibrotic scar tissue. In each row, the real image, the conditioning mask, the diffusion-generated image, and the GAN-generated image are arranged side by side, facilitating direct qualitative comparison in terms of anatomical plausibility, adherence to the conditioning mask, and overall perceptual realism. Additional qualitative examples from multiple subjects and slice locations are provided in the Appendix B, Figure A1.

### 3.2. Quantitative Evaluation of Synthetic Image Quality

To complement the qualitative assessment, we conducted a systematic comparison between the diffusion model and the CoCosNet-v2 baseline using both distribution-level and image-level metrics (Table 1). Across all evaluated quantitative measures, the diffusion-based approach consistently outperformed the GAN-based baseline.

At the distribution level, the diffusion model attained substantially lower FID (10.35 vs. 384.68) and KID values (0.0071±0.0047 vs. 0.5264±0.0269), indicating a markedly closer correspondence between the generated and reference image distributions. At the image level, the diffusion model also achieved higher SSIM (0.2491 vs. 0.1544) and PSNR (15.39 dB vs. 12.95 dB), alongside a lower LPIPS (0.2665 vs. 0.4728), consistent with enhanced structural fidelity and perceptual similarity. Furthermore, the diffusion model produced a higher Inception Score (3.58 vs. 1.29), suggesting improved sample quality and diversity relative to the GAN baseline.

Collectively, these quantitative findings provide additional evidence that diffusion-based synthesis outperforms exemplar-guided GAN-based generation for left ventricular LGE-CMR image augmentation.

As an additional qualitative validation, we conducted an expert visual realism assessment. A single CMR specialist evaluated a mixed set of real, diffusion-generated, and GAN-generated images using a 0–5 ordinal realism scale, where 0 denoted an unequivocally artificial image, and 5 denoted an image perceived as fully realistic. The assessment comprised 124 images in total: 32 real images, 43 diffusion-generated images, and 49 GAN-generated images. Images were presented in a randomised sequence, and the evaluator was blinded to the origin of each image. Real images received consistently high scores, with a mean score of 4.69±0.82 and a median of 5. Diffusion-generated images exhibited a highly similar score distribution, with a mean of 4.60±1.09 and a median of 5. In contrast, GAN-generated images were rated substantially lower, with a mean score of 1.39±1.58 and a median of 0. High-realism ratings, defined as scores of 4 or 5, were assigned to 90.6% of real images and 95.3% of diffusion-generated images, but only 12.2% of GAN-generated images.

Although this assessment was performed by a single expert reader and should therefore be regarded as qualitative supportive evidence rather than a definitive quantitative analysis, the findings suggest that diffusion-generated images were perceived as markedly more realistic than GAN-generated images and visually more similar to real CMR data.

### 3.3. Noise-Fidelity Analysis

Cross-set comparisons (REAL vs. DM, REAL vs. GAN, DM vs. GAN) were performed as paired Wilcoxon signed-rank tests on per-slice triples sharing the ground-truth mask (n=5591 slices from 639 patients), with Bonferroni correction applied within each metric family across the three pairwise contrasts. The choice of per-slice rather than per-patient pairing reflects the slice-wise conditioning of both generators: each synthesised slice constitutes an independent sample from the corresponding generator, conditional on a single mask, so aggregation to per-patient medians would discard information and reduce statistical power (full justification in the Appendix C.1). Bonferroni correction was adopted for this analysis in preference to the Holm–Bonferroni procedure used elsewhere in the manuscript because only three pairwise contrasts are evaluated per metric family; under this modest comparison count, the conservativeness penalty of Bonferroni is small, while its uniform stringency provides a more demanding test of the noise-fidelity findings. Rank-biserial correlation *r* is reported as the effect size alongside corrected *p*-values, since the cohort size saturates *p* numerically. A within-patient resampling sensitivity analysis (1000 iterations, one slice per patient per iteration; Appendix C.5 and Figure A6) provides a secondary check against pseudoreplication. Three methodological choices ensure symmetric treatment of REAL, DM, and GAN: the extra-myocardial tissue mask used for the wavelet-MAD c estimate is computed once per slice from the REAL image and reused for the DM and GAN pairings (Appendix C.2); the power spectral density is computed on the high-pass residual of each image with mask-autocorrelation bias correction, so that the spectral comparison isolates the noise component rather than mixing noise with low-frequency tissue structure (Appendix C.6); and the local-σ used in the σ(μ) analysis is computed on a detrended image rather than on the raw intensity, so that within-window signal gradients do not inflate the measured slope (Appendix C.3). Full methodological details, including the justification for per-slice pairing, the $\hat{\sigma }$ estimator, and the short-range correlation metric, are provided in the Appendix C.1, Appendix C.2, Appendix C.3, Appendix C.4, Appendix C.5, Appendix C.6.

#### 3.3.1. Validation of the Noise Model for This Cohort

**Figure A2:**
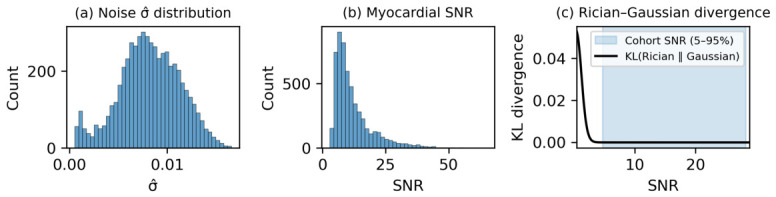
Validation of the moment-matched Gaussian approximation to Rician noise for the present cohort. (**a**) Distribution of per-image noise standard deviation $\hat{\sigma }$ estimated by wavelet MAD (Daubechies-4, diagonal detail coefficients, median/0.6745) on the extra-myocardial tissue region (Appendix C.2); values are reported on a [0,1]-scaled intensity scale. The distribution is centred at approximately 0.008. (**b**) Distribution of myocardial signal-to-noise ratio, computed as the mean myocardial intensity divided by $\hat{\sigma }$ per slice. The distribution is unimodal, with a mode near 10 and a 5–95% range of approximately 5–30. (**c**) Theoretical Kullback–Leibler divergence between a Rician distribution and a moment-matched Gaussian as a function of SNR; the shaded band marks the cohort’s empirical 5–95% SNR range, which lies entirely above the SNR threshold of ≈3, at which Rician and Gaussian become numerically indistinguishable ($\mathrm{KL}<10^{-3}$). The moment-matched Gaussian assumption invoked throughout Section 3.3 of the main text is therefore defensible at this cohort’s operating point.

Before applying any Gaussian-noise assumptions, we verify that the Gaussian approximation to the underlying Rician distribution is defensible at the operating point of the present cohort. The per-image $\hat{\sigma }$ distribution is centred at approximately 0.008 on [0, 1]-scaled images, and the myocardial signal-to-noise ratio is unimodal with a mode near 10 and a 5–95% range of approximately 5–30 (Appendix C, Figure A2a,b). Consistent with the well-established analysis of Rician noise in MR magnitude images [43], the Kullback–Leibler divergence between a Rician distribution and a moment-matched Gaussian falls below $10^{-3}$ above SNR≈3, and the cohort’s empirical SNR distribution lies entirely above this threshold (Appendix C, Figure A2c). The Gaussian approximation is therefore defensible for this cohort and justifies the analyses that follow.

#### 3.3.2. Spectral Content

**Figure 4:**
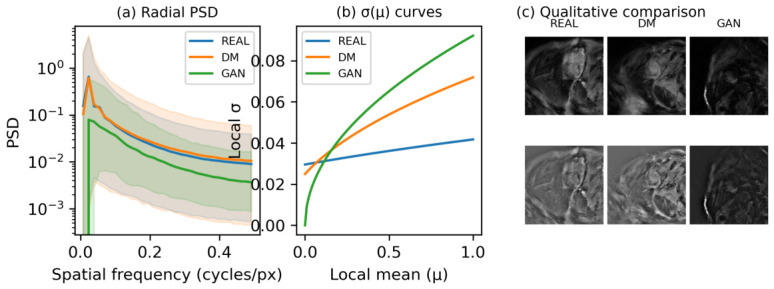
Noise-fidelity comparison of REAL, DM, and GAN image sets. (**a**) Mask-autocorrelation-corrected radial power spectral density of the high-pass-filtered myocardial ROI; solid lines show median curves across slices, shaded bands the 2.5–97.5th percentile envelopes. The DM and REAL curves overlap throughout the noise band, whereas the GAN sits approximately an order of magnitude below both over the full range. The rise below approximately 0.03 cycles/px is the signature of the Gaussian high-pass pre-filter (σ=6 px), applied identically to the three sets and therefore not a discriminating feature. (**b**) Signal-dependent variance curves σ(μ), computed per image from windowed local-mean (raw intensity) and detrended-residual local-standard-deviation estimates (Appendix C.3). The REAL curve is the shallowest, the DM is intermediate, and GAN is the steepest: REAL, DM, and GAN all preserve a sub-linear Rician-consistent shape, but differ in slope, with the DM’s slope sitting between REAL and GAN. (**c**) Qualitative same-mask comparison: two representative slices (rows), each showing REAL, DM, and GAN outputs. Statistical comparisons performed by paired Wilcoxon signed-rank tests on per-slice triples (n=5591 slices from 639 patients); pairwise effect sizes and robustness are reported in Table 2 and Table A2.

The radial power spectral density (Figure 4a) was computed on high-pass–filtered myocardial ROIs with mask-autocorrelation bias correction (Appendix C.6) so that the spectral comparison isolates the noise content of each image rather than a mixture of noise and low-frequency tissue structure. DM tracks REAL closely across the entire noise band, whereas GAN lies approximately an order of magnitude below both REAL and DM over the full supported frequency range, indicating systematic high-frequency attenuation rather than a mere distortion of shape. Quantified as per-image log-PSD distance to the median REAL PSD (Table 2, row 1), the DM-vs-GAN contrast yields median distances of 0.19 for DM and 0.90 for GAN, with rank-biserial r=−0.60 ($p_{\mathrm{corr}}<10^{-100}$, n=5537) and robustness of 100% across within-patient resamples. The REAL-vs-DM and REAL-vs-GAN rows of this metric are not defined (a reference image’s distance to its own set is identically zero) and are recorded as not applicable; the DM-vs-GAN contrast is therefore the informative measurement in this row of Table 2. The shared low-frequency roll-off visible below approximately 0.03 cycles/px in all three curves reflects the Gaussian high-pass pre-filter (σ=6 px) applied identically to REAL, DM, and GAN; the comparison therefore remains fair across methods, but the rise into this band should be read as a pre-filter artefact rather than as a spectral peak.

This pattern is compatible with the DDPM’s training objective, which requires the network to predict the noise added at each forward diffusion step: because the noise-prediction target is defined relative to noise-corrupted versions of real images, the spatial covariance of the scanner’s noise is implicitly represented in the trained model. GAN’s approximately uniform ∼10× deficit across the noise band, by contrast, is consistent with the behaviour of upsampling-based generators, which tend to suppress high-frequency content in minimising adversarial and perceptual losses, without an analogous mechanism for reproducing the data’s spectral content. Spectral matching alone, however, does not imply reproduction of higher-order noise structure, which we examine next.

#### 3.3.3. Distributional Shape

The kurtosis of high-pass-filtered myocardial intensity (Appendix C, Figure A4; Table 2) has a median of −0.43 for REAL, −0.31 for DM, and +1.00 for GAN. The DM-vs-GAN effect is r=−0.78, the REAL-vs-GAN effect r=−0.86 (both 100% robust), while the REAL-vs-DM offset is small but statistically detectable (r=−0.21, 99.1% robust; Appendix C, Table A2). High-pass skewness exhibits a parallel pattern: REAL near zero, DM slightly positive and close to REAL, GAN markedly right-shifted.

The strongly leptokurtic GAN distribution is characteristic of a mode-seeking generator with heavy-tailed reconstruction errors. The DDPM more closely matches the real distributional shape, with only a small residual offset relative to REAL. Together with the spectral-content result, two independent axes of noise structure (spatial-frequency content and intensity distribution) converge on the same comparative ordering: DDPM fidelity is higher than GAN fidelity on both axes. By DM-vs-GAN effect-size magnitude, kurtosis is the strongest discriminator in the present analysis (|r|=0.78), with the PSD comparison second (|r|=0.60).

#### 3.3.4. Signal-Dependent Variance

The per-image slope of σ(μ) (Figure 4b, Table 2 row 2; n=1036 slices meeting the windowed-statistics support criterion described in the Appendix C.3) is 0.0009 for REAL, 0.0045 for DM, and 0.0085 for the GAN; in the myocardial ROI, REAL, DM, and GAN are ordered monotonically from shallowest to steepest. All three pairwise contrasts are significant at $p_{\mathrm{corr}}<10^{-9}$: REAL-vs-DM r=−0.28, REAL-vs-GAN r=−0.51, and DM-vs-GAN r=−0.23 (Appendix C, Table A2). The DM-vs-GAN contrast is robust in 94.8% of within-patient resamples, below the ≥99.9% robustness of the other three headline DM-vs-GAN contrasts, but still well above 50%; the REAL-vs-DM and REAL-vs-GAN contrasts are robust in 99.8% and 100% of resamples, respectively.

**Figure A3:**
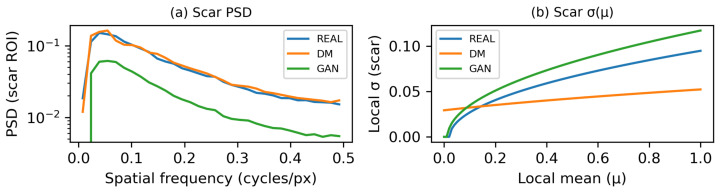
Noise-fidelity analysis restricted to the scar region of interest (n=213 slice triples meeting the ≥100 scar-voxel threshold). (**a**) Mask-autocorrelation-corrected radial power spectral density of the high-pass-filtered scar ROI for REAL, DM, and GAN (see Appendix C.6); DM tracks REAL across the full noise band, while GAN sits approximately an order of magnitude below both, mirroring the systematic high-frequency deficit observed in the myocardial analysis (main-text Figure 4a). (**b**) Signal-dependent variance σ(μ) within the scar ROI, computed with the same detrended-residual estimator used for the myocardial panel (see Appendix C.3). In the scar ROI specifically, REAL occupies an intermediate slope, DM is flatter than REAL (under-sloped), and GAN is steeper than REAL (over-sloped); both generators deviate from REAL, but in opposite directions. This scar-ROI pattern is a tissue-specific nuance of the signal-dependent-variance mechanism discussed in main-text Section 3.3.4: adversarial and perceptual losses in GAN reward signal-proportional contrast most visibly in bright scar regions, while the DDPM’s score function, trained predominantly on abundant myocardial examples, appears to under-absorb signal-dependence of noise in the rarer scar tissue class. The myocardial-ROI slope, on which the headline metric in main-text Table 2 is based, remains the primary result; the scar-ROI observation extends the noise-fidelity picture to the clinically critical tissue class.

**Figure A4:**
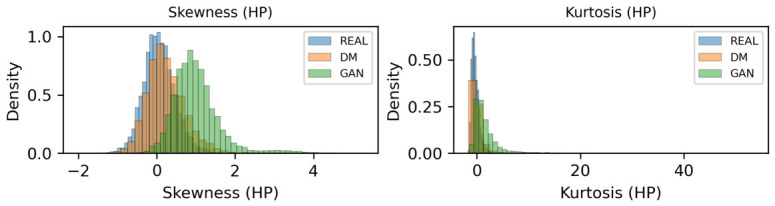
Per-image distributions of high-pass-filtered skewness (**left**) and excess kurtosis (**right**) of the myocardial ROI, for REAL, DM, and GAN image sets (n=5586 slice triples after exclusion of 5 triples with fewer than 10 myocardial voxels; moments were computed on the Gaussian high-pass residual with $\sigma_{\mathrm{hp}}=6$ px so that the distribution reflects noise rather than tissue intensity). Different colours correspond to the REAL, DM, and GAN image sets, as indicated in the figure legend. The REAL and DM distributions substantially overlap and are both centred near zero: median skewness ≈0 for REAL and slightly positive for DM; median kurtosis is −0.43 (REAL) and −0.31 (DM). The GAN distributions are right-shifted on both axes (median skewness ≈+1.0, median kurtosis +1.00) and exhibit a heavy right tail in kurtosis that extends to values above 10 (truncated in the plot). This leptokurtic signature is the visual counterpart to the Table A2 kurtosis family (row 4), in which the DM-vs-GAN effect-size magnitude (|r|=0.78) is the largest in the noise-fidelity suite.

The σ(μ) metric is computed on a detrended residual (Appendix C.3): local μ is taken from the raw image, while local σ is taken from the image minus a narrow Gaussian blur. This construction prevents within-window signal gradients from contributing to the local variance estimate, so that the measured slope reflects the noise component of σ(μ) rather than a mixture of noise and residual tissue structure. REAL images exhibit near-zero signal-dependence of noise (slope ≈ 0.001), consistent with a nearly signal-independent Gaussian-like noise model at the cohort’s SNR operating point; DM shows a small but nonzero slope (≈0.005); GAN shows a substantially larger slope (≈0.009). In the myocardial ROI (Figure 4b), the three sets preserve a sub-linear Rician-consistent shape but differ in slope, with DM sitting between REAL and GAN. In the scar ROI (Appendix C, Figure A3b), by contrast, DM and GAN depart from REAL in opposite directions (Section 3.3.6); the headline ordering reported in Table 2 therefore pertains specifically to the myocardial analysis, and the scar-ROI deviation is addressed separately below.

This pattern is compatible with the DDPM’s noise-prediction objective, which exposes the network to the scanner’s noise at every denoising step: both the functional form and the magnitude of signal-dependence of noise are therefore partially represented in the trained model. A plausible interpretation of the residual offset between REAL and DM (r=−0.28) is that the forward noise schedule contributes a modest additional signal-independent variance on top of the signal-dependent component learned from the data. GAN’s markedly steeper slope is consistent with the behaviour of adversarial and perceptual losses, which reward locally signal-proportional variance: sharpness and contrast are amplified where the generator places bright content, while background regions are flattened, without an explicit mechanism for matching the data’s near-zero signal-dependence of noise. In the myocardial ROI, all three sets preserve the Rician-consistent sub-linear shape of σ(μ); the discriminating dimension is therefore the slope, and on that dimension, DM is closer to REAL than GAN is.

#### 3.3.5. Short-Range Spatial Correlation and Robustness

**Figure A5:**
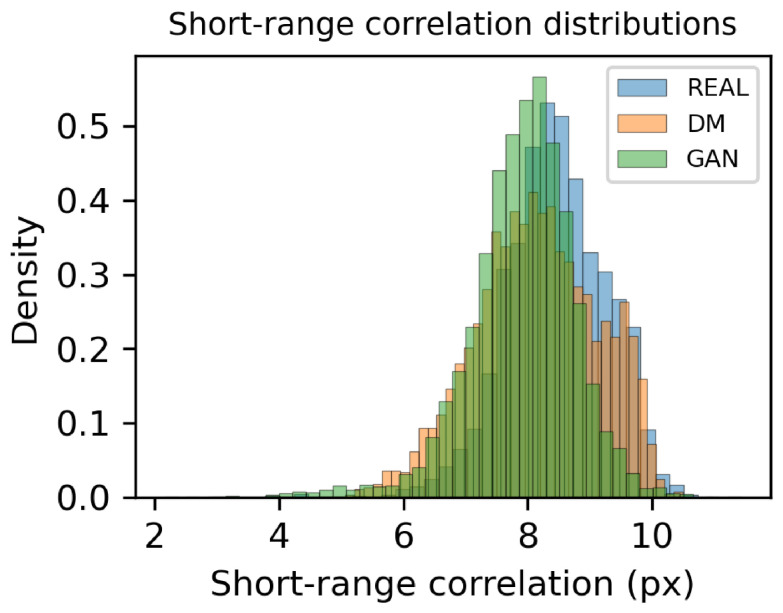
Per-image distributions of short-range spatial correlation for REAL, DM, and GAN image sets, computed as the integral of the normalised radial autocorrelation of the high-pass-filtered myocardial ROI over lags [0,10] px (Appendix C.4). Different colours correspond to the REAL, DM, and GAN image sets, as indicated in the figure legend. Larger values indicate greater spatial correlation of noise at short lags; the metric captures hardware- and reconstruction-specific noise structure at the scales most strongly influenced by *k*-space filtering, parallel imaging, and receive-coil geometry. Median values are 8.45 (REAL), 8.14 (DM), and 7.97 (GAN); n=5411 slice triples after exclusion of 180 triples with insufficient mask overlap at the 10-px lag. Pairwise effect sizes (rank-biserial) and resampling robustness are as follows: REAL vs. DM r=+0.40 (100% robust), REAL vs. GAN r=+0.53 (100% robust), DM vs. GAN r=+0.20 (99.9% robust); full test statistics are reported in Table A2. Both generators show reduced short-range correlation, with GAN deviating more severely than DM.

Short-range correlation, defined as the integral of the normalised radial autocorrelation of the high-pass-filtered myocardial ROI over lags [0,10] px (Appendix C.4), captures noise correlation at the scales most strongly shaped by *k*-space filtering, parallel-imaging reconstruction, and receive-coil geometry. Medians are 8.45 (REAL), 8.14 (DM), and 7.97 (GAN), with n=5411 slice triples after exclusion of 180 triples whose ROIs could not support the lag range with sufficient mask overlap (Appendix C, Figure A5; Table 2). Both generators underproduce short-range correlation, with GAN deviating more: REAL-vs-DM r=+0.40, REAL-vs-GAN r=+0.53, DM-vs-GAN r=+0.20, all robust in ≥99.9% of resamples.

**Figure A6:**
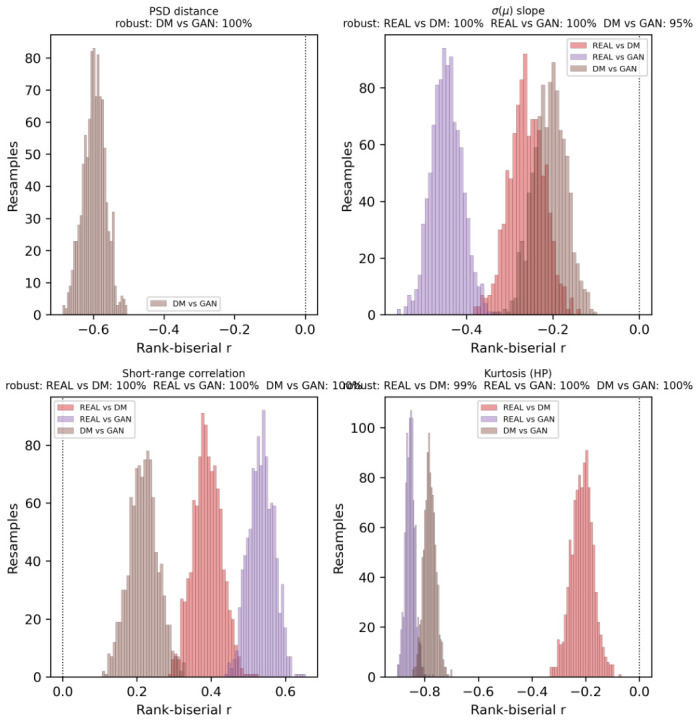
Within-patient resampling sensitivity analysis for the four headline noise-fidelity metrics (Appendix C.5). Each panel shows the distribution of rank-biserial effect sizes *r* across 1000 resampling iterations, where each iteration draws a single slice per patient (n≈639) and reruns the three paired Wilcoxon signed-rank tests with Bonferroni correction within each metric family. Different colours denote the pairwise contrasts shown in the corresponding panel legends. The subtitle of each panel reports the robustness fraction (percentage of iterations in which each pair remained significant at $p_{\mathrm{corr}}<0.05$); the dashed vertical line marks r=0 for reference. The PSD-distance panel shows only the DM-vs-GAN histogram, because REAL-vs-X contrasts are not defined for that metric (the PSD distance of each REAL image to its own reference is identically zero; see Appendix C.6). Overlapping histogram content does not affect scientific interpretation, because the distributions are summarised by the reported robustness fractions and rank-biserial effect-size ranges. Headline DM-vs-GAN robustness values: PSD distance 100%; σ(μ) slope 94.8%; short-range correlation 99.9%; high-pass kurtosis 100%. The narrowest DM-vs-GAN margin is the σ(μ)-slope contrast at 94.8%, consistent with the comparatively smaller rank-biserial effect (r=−0.23 in the full-*n* test) for that metric; the other three headline DM-vs-GAN contrasts cluster at the resampling ceiling. All three REAL-vs-GAN contrasts for metrics where they are defined (σ(μ) slope, short-range correlation, high-pass kurtosis) are robust in 100% of resamples, strengthening the uniform-failure interpretation of the GAN baseline developed in Section 3.3 of the main text.

The mechanism is consistent with the picture developed above. The DDPM represents spatial noise structure through the noise-prediction objective, but does so imperfectly at the shortest lags because the forward process injects pixel-independent noise at every step, biasing the learned density towards mild under-correlation at the pixel scale. GAN’s more severe deviation is consistent with a fixed spatial smoothing imposed by its upsampling layers, irrespective of the true short-range correlation structure of the training data. The DDPM’s residual under-correlation (r=+0.40 vs. REAL) is the most visible imperfection uncovered by the present analysis; it is qualitatively aligned with but substantially smaller than GAN’s deviation (r=+0.53). All four headline DM-vs-GAN findings survive the within-patient resampling sensitivity analysis (Appendix C.5 and Figure A6) at ≥94.8% robustness, with three of the four at ≥99.9%; the σ(μ)-slope contrast has the narrowest robustness margin (94.8%), while PSD distance and high-pass kurtosis sit at 100% robustness.

#### 3.3.6. Qualitative Inspection and the Scar Region

The quantitative findings are visually apparent in mask-matched comparisons (Figure 4c): the high-pass residuals of REAL and DM are structurally similar in texture and granularity, while the GAN residuals are visibly smoother and lack fine-scale variation, in line with both the high-frequency PSD attenuation and the over-smoothed short-range correlation. Restricting the analysis to the scar ROI (n=213 slices meeting the ≥100-voxel threshold; Appendix C, Figure A3) reproduces the same spectral pattern in the clinically critical tissue class: the DM scar PSD tracks REAL across the full frequency band, while GAN attenuates. The scar σ(μ) panel (Appendix C, Figure A3b) shows a nuance worth reporting: in the scar ROI, the DM slope is lower than the REAL slope, while GAN slope is markedly higher than both; both generators deviate from REAL but in opposite directions. The myocardial-ROI slope, on which the Table 2 headline is based, remains the primary result; the scar-ROI observation is reported here as a tissue-specific nuance of the same mechanism.

#### 3.3.7. Synthesis: Noise Fidelity as a Mechanistic Basis for the Augmentation Gap

The four noise-fidelity axes examined in the preceding subsections can be read together as a comparative profile of how the two generators represent the scanner’s noise distribution. The DDPM is trained to predict the noise added at each forward diffusion step; because this target is defined relative to noise-corrupted versions of real images, properties of the scanner’s noise are implicitly represented in the trained model. Each empirical finding maps onto a specific aspect of this representation: spectral content (Section 3.3.2) maps to spatial covariance, where DM tracks REAL to within log-PSD distance 0.19 while GAN lies ∼10× below in absolute PSD magnitude (DM-vs-GAN r=−0.60); distributional shape (Section 3.3.3) maps to higher-order moments of the noise density, where the DM’s kurtosis median (−0.31) is close to REAL’s (−0.43) and GAN is leptokurtic (+1.00; DM-vs-GAN r=−0.78); signal-dependent variance (Section 3.3.4) maps to the coupling between local signal level and local noise magnitude, where REAL, DM, and GAN are monotonically ordered in the myocardial ROI from shallowest to steepest slope (0.001<0.005<0.009; DM-vs-GAN r=−0.23); and short-range correlation (Section 3.3.5) maps to hardware- and reconstruction-specific spatial correlations, partially reproduced by the DDPM (r=+0.40 vs. REAL) and more severely distorted by GAN (r=+0.53; DM-vs-GAN r=+0.20). Therefore, the GAN baseline departs from REAL on all four axes (lower spectral magnitude, right-shifted distributional shape, steeper signal-dependent variance, more severe short-range smoothing); this pattern is consistent with the behaviour of adversarial and perceptual losses, which reward locally signal-proportional contrast and penalise high-frequency content that does not serve the discriminator, without an explicit mechanism for enforcing the data’s noise covariance, near-Gaussian shape, or near-signal-independent magnitude.

This account provides a plausible mechanistic basis for the segmentation gap reported in Section 3.5 and Section 3.5.1, in which the nnU-Net trained with DM-augmented data outperforms the GAN-augmented variant on fibrosis Dice. DM-augmented training exposes the network to images whose noise structure matches the real test distribution across all four axes examined, so the network does not learn to rely on features that are augmentation artefacts; GAN-augmented training, by contrast, exposes it to images with structurally different noise and (Section 3.3.8) systematically different myocardial contrast, both of which are expected to induce a train–test distribution shift. We frame this as a mechanistic hypothesis consistent with the observed performance gap rather than as a proven causal claim: disentangling the relative contributions of noise fidelity and intensity calibration would require an experiment with intensity-matched GAN outputs and is identified as future work.

#### 3.3.8. Intensity Calibration: A Second Mechanistic Factor

**Figure 5:**
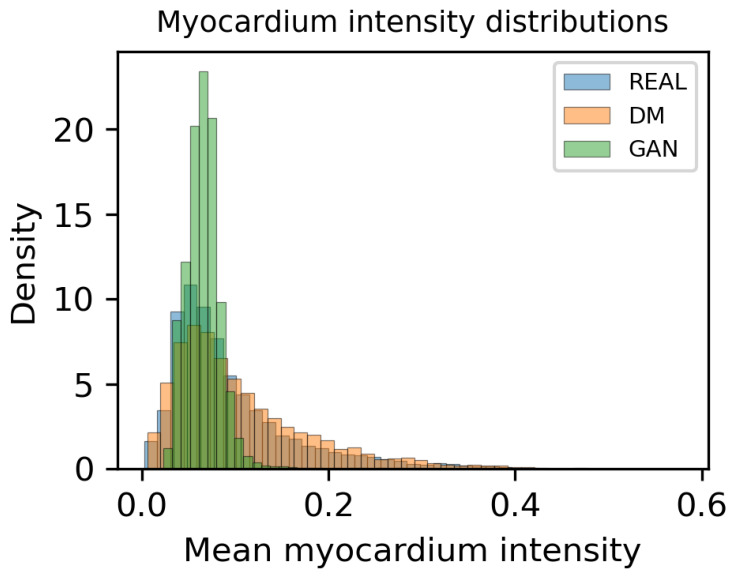
Distributions of per-image mean myocardial intensity for REAL, DM, and GAN image sets on a common axis (n=5591 slices from 639 patients). REAL peaks at approximately 0.11, DM at approximately 0.12, and GAN at approximately 0.06: GAN produces systematically dimmer myocardium, while DM is very slightly brighter than REAL. This contrast and calibration observation is distinct from the noise-fidelity analysis (see Section 3.3.8) and is presented here as a second, independent mechanistic factor potentially contributing to the downstream segmentation gap.

Beyond noise structure, the generators differ in myocardial intensity calibration (Figure 5): the per-image mean myocardial intensity peaks at approximately 0.11 for REAL and 0.12 for DM, but at approximately 0.06 for GAN. This contrast deficit is a calibration issue distinct from noise fidelity and likely contributes independently to the observed segmentation gap.

Collectively, the analyses presented here support three conclusions. First, the mask-conditioned DDPM reproduces the scanner noise structure across all four axes examined—spectral content, distributional shape, signal-dependent variance, and short-range correlation—with DM-vs-GAN effect-size magnitudes ranging from |r|=0.20 (short-range correlation) to |r|=0.78 (high-pass kurtosis), $p_{\mathrm{corr}}<10^{-9}$ in every headline contrast, and within-patient resampling robustness of 94.8% to 100% (three of four headline contrasts at ≥99.9%). The DDPM is not a perfect reproduction of real images: residual deviations from REAL appear as a modest offset in kurtosis (r=−0.21), a small σ(μ)-slope offset (r=−0.28), and a moderate short-range under-correlation (r=+0.40). However, these imperfections are circumscribed, quantified, and uniformly smaller in magnitude than the GAN’s deviations on every axis. All claims are scoped to the single scanner of the present cohort, with multi-scanner validation identified as follow-up work. Second, the analysis contributes on three fronts: empirically, it provides, to our knowledge, the first demonstration that a mask-conditioned DDPM reproduces scanner-specific noise statistics in LGE-CMR; conceptually, it maps each empirical finding onto a specific property of the scanner’s noise distribution that the DDPM’s noise-prediction objective exposes during training; and methodologically, it offers a four-axis noise-fidelity protocol (spectral content, signal-dependent variance, short-range correlation, and distributional shape, with rank-biserial effect sizes and within-patient resampling robustness) as a reusable template for evaluating generative models in medical imaging. Third, noise fidelity and intensity calibration together provide a mechanistic basis for the augmentation gap between DDPM- and GAN-based synthetic data reported earlier in this manuscript, strengthening the case for diffusion-based augmentation as the preferred strategy for LGE-CMR fibrosis segmentation under single-scanner conditions.

### 3.4. Real-Only Reference Performance

We first establish a reference model trained exclusively on real data by fitting nnU-Net on all available real training cases (n=1230), achieving Dice coefficients of 0.8937 for myocardium and 0.2910 for scar (Table 3). While myocardial delineation is consistently accurate, the substantially lower Dice score for scar reflects the well-documented difficulty of fibrosis segmentation in LGE imaging, where the target class is sparse and exhibits pronounced morphological heterogeneity. Comparable constraints have been documented in other CMR-based investigations. For example, Abdulkareem et al. [44] assessed scar prediction on 272 CMR examinations from the Barts BioResource (resulting in 722 cine—mask pairs after quality control) and reported a mean scar Dice coefficient of 0.20±0.17 (median 0.14), despite employing a scar-weighted Dice loss. These findings underscore the inherent ambiguity and pronounced class imbalance associated with myocardial scar delineation. Scar precision (0.3974) and recall (0.4332) further indicate that, even in this full-data regime, the model still encounters a non-trivial trade-off between false-positive and false-negative predictions, inherent to the high variability of the acquisition signal. The corresponding scar HD95 is 21.1866 mm, providing a complementary boundary-based reference for the analyses below.

To quantify the dependence on the amount of annotated data, we trained nnU-Net on progressively larger subsets of real data only (n=100 to 600; Table 3). Myocardium segmentation performance remained numerically stable across all configurations (Dice coefficient 0.8835–0.8937), indicating that, under the adopted ROI standardisation protocol, the myocardium segmentation task can be effectively learned from a comparatively limited number of labelled cases. Nevertheless, relative to the 1230-case baseline, significant paired differences in myocardium Dice were still observed for several reduced-data configurations.

In contrast, scar segmentation exhibited a pronounced performance scaling with the number of real training samples. Scar Dice increased from 0.1731 at n=100 to 0.2951 at n=600, corresponding to an absolute improvement of +0.1220. Notably, the performance observed at n=600 is numerically comparable to that of the real-only reference model trained on the full dataset, although the paired comparison still indicates a significant difference in scar Dice versus the 1230-case baseline. The precision-recall characteristics further indicate that the lowest-data regime is predominantly recall-limited: at n=100, scar precision is relatively high (0.4564), whereas recall is very low (0.1730), consistent with systematic under-detection of scar tissue. As the number of real training cases increases, scar recall generally improves (up to 0.4090 at n=500), reflecting enhanced sensitivity to small lesions, while precision shows non-monotonic variations, consistent with the intrinsic signal ambiguity of scar boundaries in LGE imaging. A similar trend is observed for HD95, which decreases from 29.5233 at n=100 to 20.4522 at n=500, before increasing again at n=600 (24.9378), further supporting a non-monotonic precision–recall and boundary-quality trade-off. Statistical analysis confirms that most significant differences relative to the full-data baseline are concentrated in the scar class and in the lower training-set regimes, whereas the 500-case configuration is among the closest to the full-data reference overall. Additional class-wise results, including HD95, precision, recall, and the corresponding paired statistical comparisons versus the 1230-real reference model, are provided in the Appendix D, Table A3 and Table A4.

### 3.5. Diffusion-Based Synthetic Augmentation

To prevent data leakage, the diffusion-based generator is first pretrained on 600 non-overlapping cases and subsequently employed as a fixed stochastic sampling mechanism for all augmentation experiments. We then evaluate diffusion-based synthetic augmentation under three distinct experimental regimes: (i) an extreme low-data setting comprising 100 real training cases (R100), (ii) an intermediate-data setting comprising 300 real training cases (R300), and (iii) a higher-data setting comprising 600 real training cases (R600), while maintaining the validation and test sets unchanged to ensure a consistent evaluation benchmark.

**Figure 6:**
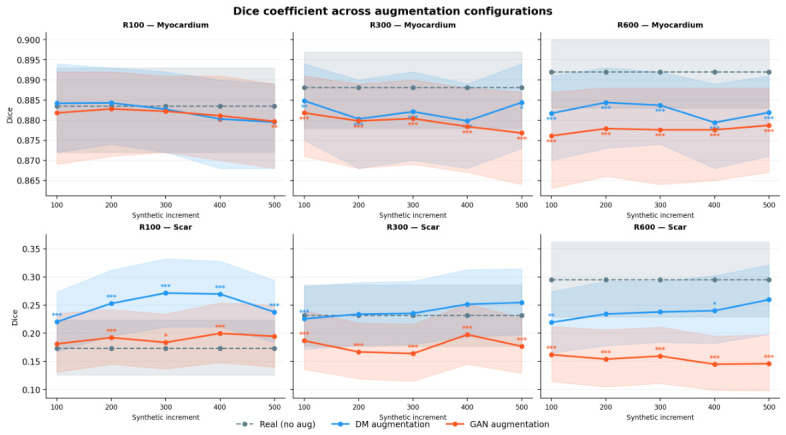
Dice coefficient as a function of synthetic increment size for Myocardium (**top row**) and Scar (**bottom row**) across the three real dataset sizes (columns: R100, R300, R600). Different coloured lines distinguish the augmentation strategies, and the corresponding shaded regions represent the 95% bootstrap confidence intervals around the mean Dice values. The dashed grey line denotes the no-augmentation baseline for the corresponding real-data regime. Asterisks placed above individual points indicate a statistically significant difference relative to the corresponding no-augmentation baseline at the same dataset size, based on the Holm-corrected Wilcoxon signed-rank test: * p<0.05, ** p<0.01, *** p<0.001.

Figure 6 summarises the trajectories of the Dice coefficients for scar and myocardium across all three training regimes, presenting the mean ± 95% confidence interval for the no-augmentation baseline, diffusion model (DM), and generative adversarial network (GAN) conditions as a function of the size of the synthetic data increment. This panel provides the global context for the subsequent regime-specific analysis: under R100, the DM curve increases and attains its maximum at an increment of 300, remains approximately constant under R300, and decreases under R600, whereas the GAN curve exhibits a downward divergence in the R300 and R600 regimes.

In the low-data regime, diffusion-based augmentation yields substantial improvements in scar segmentation relative to the R100 baseline (Table 4 vs. Table 3). Scar Dice increases from 0.1731 (100 real cases only) to a maximum of 0.2714 for the R100+300 DM synthetic configuration, corresponding to an absolute gain of +0.0983 (approximately +56.8% relative). This improvement is predominantly driven by enhanced recall: scar recall rises from 0.1730 to 0.3625 (+0.1895, i.e., more than a two-fold relative increase), whereas precision decreases moderately from 0.4564 to 0.4183. The substantial improvement in recall indicates that the diffusion model effectively captures and reconstructs the ambiguous and noise-corrupted signal characteristics of scar tissue. By training on these statistically noisy synthetic samples, the segmentation network attains increased robustness to the low-SNR conditions commonly encountered in real clinical acquisitions. These results indicate that, in the most annotation-limited regime, diffusion-based augmentation primarily alleviates conservative under-segmentation, enhancing sensitivity at the expense of a moderate increase in false-positive predictions. A similar trend is observed for HD95, with scar HD95 decreasing from 29.5233 in the R100 baseline to 23.4964 in the R100+300 DM configuration, and reaching its lowest value at R100+200 DM (21.9224). Several of these improvements for the scar class, including Dice, recall, and HD95, are statistically significant relative to the R100 baseline.

**Figure 7:**
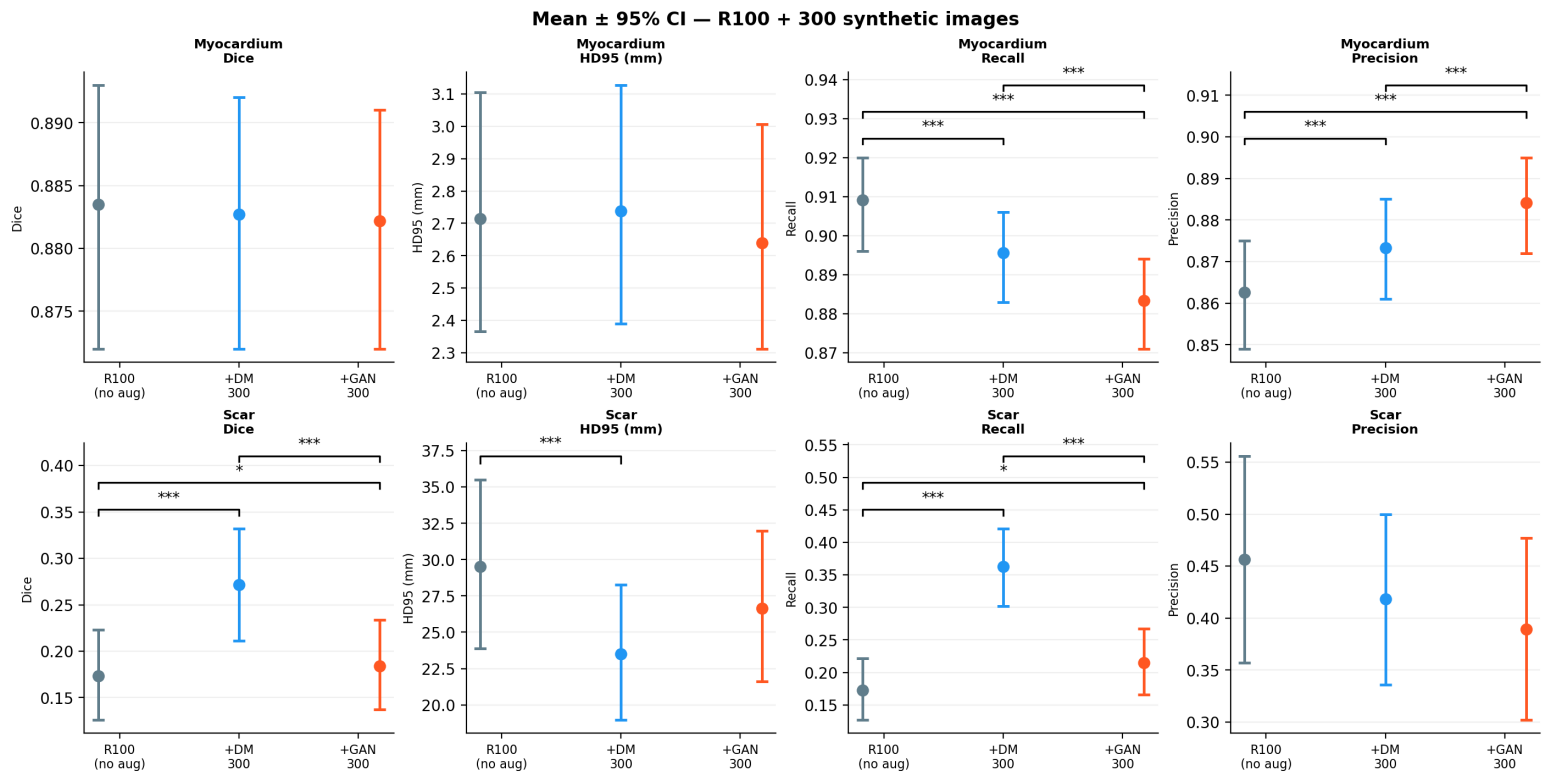
Mean ± 95% bootstrap confidence intervals for Dice, HD95, recall, and precision for the R100+300 DM configuration, reported for Myocardium (**top row**) and Scar (**bottom row**). Different colours indicate the compared training configurations shown in each panel. Statistical significance is indicated by brackets corresponding to Holm-adjusted pairwise Wilcoxon tests. Significance codes, where present, are: * p<0.05, ** p<0.01, and *** p<0.001. The absence of a bracket denotes p>0.05 following multiple-comparison correction.

Figure 7 provides a magnified view of the peak-performing configuration (R100+300DM synthetic images), depicting the mean and 95% bootstrap confidence intervals for all four primary metrics across both tissue types. The non-overlapping confidence intervals for scar Dice and recall between the no-augmentation baseline and DM corroborate the robustness of the improvements reported above. In contrast, the overlapping confidence intervals for precision, in line with the non-significant change in precision (p=0.156), visually support the interpretation that any potential reduction in precision is modest and statistically uncertain rather than systematic. Furthermore, the substantial overlap of the GAN confidence intervals for scar Dice and HD95 with those of the baseline, even at this augmentation level, anticipates the weaker and less consistent performance gains from GAN-based augmentation discussed in Section 3.5.1.

The impact of progressively increasing the amount of synthetic data is, however, non-monotonic. Beyond the R100+300DM configuration, additional synthetic samples do not yield further improvements: the scar Dice score remains effectively unchanged at R100+400DM (0.2696) and subsequently decreases to 0.2377 at R100+500DM. Notably, recall continues to rise, reaching 0.3884 at R100+400DM, but this increase is accompanied by a reduction in precision (0.3814), indicating a progressively more permissive decision boundary that alters the precision–recall trade-off without enhancing overall spatial overlap. This pattern is consistent with the existence of an optimal real-to-synthetic data ratio under low-data conditions; surpassing this ratio may induce distributional shifts or overemphasise scar-like signal characteristics, thereby perturbing the balance between precision and recall. Across all low-data augmentation configurations, myocardium segmentation performance remains essentially stable (Dice ≈0.88), suggesting that the beneficial effect of diffusion-based augmentation is predominantly confined to the scar class. Additional class-wise quantitative results for the $N_{\mathrm{real}}=100$ diffusion regime, including precision, recall, HD95, and full paired statistical comparisons, are provided in Appendix D, Table A5 and Table A6.

An additional ablation assessing the effect of loss weighting in the R100 diffusion regime is provided in the Appendix D, Table A7. Briefly, the weighted-loss formulation mainly improved scar Dice and recall relative to the unweighted counterpart, supporting the role of imbalance-aware optimisation in a low-data setting.

We further assess diffusion-based augmentation in an intermediate-data regime comprising R300 training cases (Table 5). Relative to the 300 baseline (scar Dice of 0.2315), the highest scar segmentation performance is achieved with the R300+500 configuration, which yields a scar Dice of 0.2545. This corresponds to an absolute improvement of +0.0230 (approximately +9.9% relative). Unlike in the low-data regime, this gain is not associated with a consistent improvement in recall, and the effects of synthetic augmentation are more limited and non-monotonic. For example, the R300+200 setting produces only a marginal increase in scar Dice to 0.2340, whereas the R300+100 setting underperforms the baseline (scar Dice of 0.2256). The best scar Dice values in this regime are observed for R300+400 and R300+500 (0.2515 and 0.2545, respectively). HD95 shows a similarly non-monotonic pattern, with modest reductions at R300+200 (22.6182) and R300+400 (22.2092) relative to the baseline (23.5770), but without a clear monotonic trend across configurations. In this regime, statistically significant improvements are more limited and are mainly observed in isolated metrics rather than as a consistent gain in scar Dice.

Across the remaining augmentation configurations, the impact of synthetic data is non-monotonic. For instance, the R300+200 setting does not substantially alter the precision–recall balance for the scar class, while the R300+300 configuration produces only a limited change (scar Dice of 0.2353). Myocardial segmentation performance remains approximately constant across all configurations (Dice ≈0.88), indicating that the observed effects are largely specific to the scar class. Additional class-wise quantitative results for the $N_{\mathrm{real}}=300$ diffusion regime, including precision, recall, HD95, and full paired statistical comparisons, are reported in the Appendix D, Table A8 and Table A9.

With a substantially larger real training dataset, diffusion-based augmentation does not confer additional benefits for scar segmentation under the fixed data split (Table 6). Across all real-to-synthetic ratios, the scar Dice coefficient remains below that of the R600 baseline (scar Dice of 0.2951; Table 6). The best-performing augmented configuration is R600+500, achieving a scar Dice of 0.2596, which still underperforms both the R600 model (absolute difference of −0.0355, corresponding to approximately −12.0% relative) and the 1230-real reference baseline (scar Dice of 0.2910; Table 3). These findings indicate that, in this higher-data regime, incorporating synthetic samples does not translate into improved overlap-based performance for scar segmentation. This conclusion is also supported by HD95: although scar HD95 is slightly lower for some augmented settings, such as R600+200 (23.6203) and R600+300 (23.2557), than for the R600 baseline (24.9378), these reductions are modest and are not accompanied by improvements in scar Dice.

The precision-recall profile further suggests a redistribution of errors rather than a uniform performance degradation. Relative to the R600 configuration (precision 0.4691, recall 0.3492), the R600+500 setup slightly reduces scar precision to 0.4548 and reduces scar recall to 0.3012, consistent with the lower scar Dice observed in this regime. This dependence on the real-to-synthetic ratio is further evidenced by the non-monotonic performance trends observed across configurations (Table 6). Myocardial segmentation performance remains generally stable but exhibits a modest decline relative to the R600 baseline (Dice 0.8920 vs. 0.8794–0.8844 across augmented configurations). No statistically significant improvement is observed for the scar class in this regime. Additional class-wise quantitative results for the $N_{\mathrm{real}}=600$ diffusion regime, including precision, recall, HD95, and full paired statistical comparisons, are provided in the Appendix D, Table A10 and Table A11.

#### 3.5.1. GAN-Based Synthetic Augmentation (CoCosNet-v2)

To enable a controlled comparison, we preserve the nnU-Net training protocol and the fixed data partition identically across all experimental conditions, varying exclusively the source of synthetic images (DDPM-generated versus CoCosNet-v2-generated samples). We assess exemplar-guided GAN-based augmentation (CoCosNet-v2) under the same low-, intermediate-, and higher-data regimes as those employed for diffusion-based augmentation (Table 7, Table 8 and Table 9 vs. Table 4, Table 5 and Table 6).

Under the R100 regime, the optimal GAN-based augmentation setting is R100+400 GAN, yielding a scar Dice coefficient of 0.1998 (Table 7). By comparison, diffusion-based augmentation attains a substantially higher optimum in this data-constrained scenario: the best diffusion configuration (R100+300) achieves a scar Dice coefficient of 0.2714 (Table 4). Overall, diffusion augmentation surpasses GAN augmentation by +0.0716 Dice (corresponding to an approximately +35.8% relative improvement over the GAN optimum). The corresponding precision–recall characteristics indicate that diffusion attains this gain through a more favourable balance between sensitivity and precision: at their respective optima, diffusion achieves a scar recall of 0.3625 versus 0.2696 for GAN, and a scar precision of 0.4183 versus 0.3390 for GAN. This observed performance disparity indicates that diffusion models yield a more stable and faithful characterisation of the underlying pathological data distribution than the GAN-based translation baseline. A similar trend is observed for HD95, with diffusion achieving lower scar HD95 values than GAN in this regime (e.g., 23.4964 at R100+300 for diffusion versus 25.6495 at R100+400 for GAN, with the lowest GAN scar HD95 observed at 25.1972 for R100+200). Although several GAN configurations show statistically significant improvements over the R100 baseline for scar Dice, recall, and HD95, diffusion remains consistently superior.

This performance disparity is further illustrated in Figure 6 (R100 column, Scar row). Across all increment levels, the curve corresponding to the GAN-based augmentation exhibits only a minimal increase and remains consistently and substantially lower than the curve associated with the diffusion model. This pattern indicates that the superiority of the diffusion-based approach is not restricted to a particular increment value, but is maintained across the entire spectrum of synthetic data volumes examined.

At the increment level at which DM attains its maximum effect (R100+300), the divergence between the two approaches is also evident in Figure 7. Specifically, the confidence intervals of DM for scar Dice and HD95 are clearly separated from those of the baseline, whereas the corresponding GAN confidence intervals exhibit substantial overlap with the no-augmentation condition. This pattern suggests that, although the GAN configuration yields a statistically significant improvement, it results in only a limited practical separation from the baseline when examined at the level of individual metrics. Additional class-wise quantitative results for the $N_{\mathrm{real}}=100$ GAN regime, including precision, recall, HD95, and full paired statistical comparisons versus both the corresponding real-only baseline and diffusion-based augmentation, are provided in the Appendix D, Table A12 and Table A13.

With R300 training cases, diffusion-based augmentation continues to demonstrate a marked advantage over GAN-based augmentation (Table 5 and Table 8). The best-performing diffusion configuration is R300+500, yielding a scar Dice coefficient of 0.2545, whereas the optimal GAN configuration (R300+400) achieves a Dice coefficient of 0.1976. This corresponds to an absolute Dice improvement of +0.0569 in favour of diffusion, which represents an approximate +28.8% relative gain over the optimal GAN performance. At their respective optima, diffusion exhibits slightly lower scar precision than GAN (0.4307 vs. 0.4370), but substantially higher recall (0.3022 vs. 0.2172). Overall, diffusion attains the higher scar Dice because the gain in recall outweighs the modest precision advantage of GAN. HD95 also favours diffusion in this regime: scar HD95 decreases to 22.2092 at R300+400 for diffusion, whereas the corresponding GAN configuration reaches 26.2450. Statistically significant improvements for GAN in this regime are limited and mainly confined to isolated metrics rather than to a consistent gain in scar Dice. Additional class-wise quantitative results for the $N_{\mathrm{real}}=300$ GAN regime, including precision, recall, HD95, and full paired statistical comparisons versus both the corresponding real-only baseline and diffusion-based augmentation, are reported in the Appendix D, Table A14 and Table A15.

In the R600 setting, diffusion-based augmentation remains superior to GAN-based augmentation, although both approaches underperform the R600 reference with respect to scar overlap (Table 6 and Table 9). The best-performing diffusion configuration is R600+500 (scar Dice = 0.2596), whereas the best-performing validated GAN configuration is R600+100 (scar Dice = 0.1621). Consequently, diffusion surpasses GAN by an absolute margin of +0.0975 Dice, corresponding to an approximate +60.1% relative improvement over the best-performing GAN setup. The associated error characteristics also differ systematically: GAN-based models can attain comparatively high precision in certain configurations (e.g., 0.5822 at R600+200) but exhibit consistently low recall (0.1249–0.1523 across the higher-performing settings). In contrast, diffusion-based models achieve substantially higher recall (up to 0.3012 at R600+500) while maintaining competitive precision, resulting overall in superior segmentation overlap. The same pattern is reflected in HD95, where GAN-based augmentation remains worse than both the real-only baseline and diffusion-based augmentation across all evaluated settings. Moreover, no statistically significant improvement is observed for the scar class for GAN in this regime, except for an isolated increase in scar precision at R600+200. Additional class-wise quantitative results for the $N_{\mathrm{real}}=600$ GAN regime, including precision, recall, HD95, and full paired statistical comparisons versus both the corresponding real-only baseline and diffusion-based augmentation, are provided in the Appendix D, Table A16 and Table A17.

**Figure 8:**
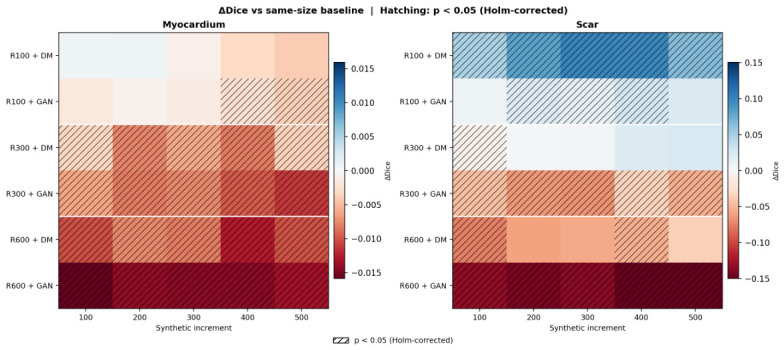
ΔDice values relative to the corresponding same-size, non-augmented baseline are reported for all configurations. Each row represents a diffusion model (DM) and generative adversarial network (GAN)-based augmentation within a given real dataset size (R100, R300, R600), while each column denotes the size of the synthetic data increment. Blue shading indicates an improvement, and red shading indicates a deterioration in performance. Hatched cells denote statistically significant differences after Holm correction (p<0.05). White grid lines delineate groups of configurations sharing the same real dataset size.

The regime-dependent performance disparity between DM and GAN is summarised in Figure 8, which depicts ΔDice values relative to the same-size, no-augmentation baseline for all 30 configurations and both tissue types. Here, ΔDice denotes the difference between the Dice score of an augmented model and that of the corresponding real-only baseline trained with the same number of real cases. A clear asymmetry between the two augmentation strategies is evident: rows corresponding to DM exhibit positive ΔDice values (blue) at R100 and values close to zero at R300, whereas rows corresponding to GAN are consistently negative (red) at R300 and R600, with nearly all entries attaining statistical significance. This spatial pattern integrates the regime-specific findings described above into a single visual representation and clarifies the practical implications: DM-based augmentation is advantageous in low-data regimes and approximately performance-neutral at higher data availability, whereas GAN-based augmentation becomes deleterious once a moderately sized real dataset is available.

#### 3.5.2. Repeated-Seed Robustness Analysis

**Figure 9:**
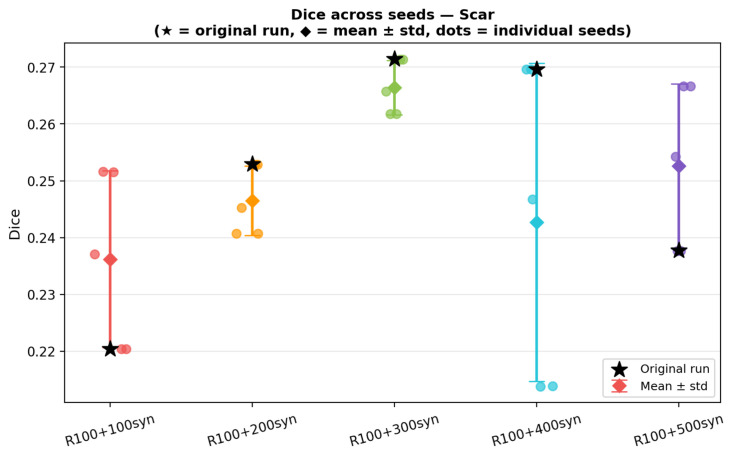
Seed-stability analysis of diffusion-based data augmentation in the low-data regime. Dice similarity coefficient (DSC) values for scar segmentation are reported across five independent random seeds for each synthetic-data configuration. Different colours denote the different synthetic-data configurations evaluated. The results demonstrate that the initially reported single-run scar DSC values are broadly representative of the overall comparative performance pattern observed across augmentation conditions, with the R100+300 syn configuration consistently showing the highest performance.

To assess robustness with respect to stochastic initialisation, additional repeated-seed experiments were performed for diffusion-based data augmentation in the low-data regime ($N_{\mathrm{real}}=100$). As summarised in Table 10, myocardium segmentation exhibited very low variability across five independent runs, whereas scar segmentation demonstrated low-to-moderate variability overall, with the largest variability observed for the R100+400 condition. The best-performing diffusion configuration, R100+300, yielded a scar DSC of 0.2664±0.0048 (coefficient of variation = 1.8%), reflecting high reproducibility. This result is further corroborated by Figure 9, which depicts the seed-wise scar DSC values for each diffusion condition. Collectively, these findings suggest that the originally reported single-run values are broadly representative of the overall comparative behaviour, with R100+300 consistently emerging as the best-performing configuration across repeated runs, although a full multi-seed evaluation of all experimental configurations was precluded by computational constraints.

## 4. Conclusions

The experimental findings elucidate several aspects of the role of generative augmentation in LGE-CMR segmentation. First, myocardium segmentation appears comparatively robust and demonstrates early performance saturation across training configurations using only real data, whereas scar segmentation shows pronounced data dependence, with substantial performance gains as the number of real training cases increases. This discrepancy is consistent with the greater spatial sparsity, morphological heterogeneity, and interpretative ambiguity of fibrotic tissue in LGE-CMR.

Second, under matched downstream training conditions, diffusion-based augmentation consistently outperforms GAN-based synthesis across all evaluated regimes. The performance advantage is most prominent in the extreme low-data setting, where diffusion-based augmentation yields substantially higher scar DSC and recall compared with the GAN baseline. Similar trends are observed at intermediate and higher real-data budgets, although the absolute benefit of incorporating synthetic samples diminishes as the volume of real training data increases.

Third, the impact of synthetic augmentation is non-monotonic and depends critically on the real-to-synthetic data ratio. In the most annotation-limited regime, diffusion-based augmentation primarily enhances sensitivity to scar, whereas in intermediate regimes, the optimal configurations also improve spatial overlap by achieving a more favourable precision–recall trade-off. However, once a sufficiently large pool of real training data is available, synthetic augmentation does not confer additional improvements over the real-only baseline, even though diffusion-based synthesis remains superior to GAN-based augmentation.

Fourth, the four-axis noise-fidelity analysis (spectral content, signal-dependent variance, short-range spatial correlation, and distributional shape) demonstrates that the mask-conditioned DDPM reproduces scanner-specific noise statistics substantially more faithfully than the GAN baseline on every axis examined, with DM-vs-GAN effect-size magnitudes ranging from |r|=0.20 to |r|=0.78 and within-patient resampling robustness of 94.8% to 100%. Together with a separate intensity-calibration observation, this noise-fidelity gap provides a plausible mechanistic account for the augmentation benefit of diffusion-based over GAN-based synthetic data; we frame this account as a mechanistic hypothesis consistent with the observed performance gap rather than as a proven causal claim.

This study has several limitations. First, all images were acquired from a single source using Siemens scanners at 1.5T and 3T, and the training, validation, and test sets share a common data distribution. Consequently, the generalisability of the proposed approach to images from other vendors, acquisition protocols, or clinical centres remains uncertain. Future work will include cross-dataset evaluations to quantify robustness under heterogeneous acquisition and population conditions. Second, the potential clinical applicability of the method would be strengthened by a qualitative expert assessment of both the generated images and the resulting segmentation outputs. Such evaluation was not undertaken in the present study and should be incorporated into subsequent investigations. Third, the noise-fidelity findings are scoped to the single scanner of the present cohort and do not disentangle the relative contributions of noise fidelity and intensity-calibration mismatches to the augmentation gap; a controlled experiment with intensity-matched GAN outputs, together with multi-scanner validation, is identified as future work.

In summary, the results support mask-conditioned diffusion-based synthesis as an effective augmentation strategy for scar segmentation in annotation-limited LGE-CMR scenarios, while also indicating that its practical value requires further confirmation in broader multicentre settings with systematic expert review.

## Figures and Tables

**Table 1:** Quantitative evaluation metrics for diffusion-based (DM) and GAN-based image synthesis. Lower scores correspond to superior performance for FID, KID, and LPIPS, whereas higher scores indicate superior performance for SSIM, IS, and PSNR. PSNR values are reported in decibels (dB).

Model	FID	KID	SSIM	IS	LPIPS	PSNR (dB)
DM	10.35	0.0071 ± 0.0047	0.2491 ± 0.0694	3.5807 ± 0.1400	0.2665 ± 0.0682	15.39 ± 2.18
GAN	384.68	0.5264 ± 0.0269	0.1544 ± 0.0512	1.2859 ± 0.0188	0.4728 ± 0.0387	12.95 ± 2.06

**Table 2:** Headline noise-fidelity metrics with DM-vs-GAN statistical comparison. Medians are reported on the [0,1]-scaled intensity axis (PSD distance is mean-squared log-PSD difference, dimensionless; σ(μ) slope is the first-order coefficient of the variance-in-mean fit, units ${\left[0,1\right]}^{-1}$; short-range correlation is in pixels; kurtosis is the excess kurtosis). *p*-values are Bonferroni-corrected within the three pairwise contrasts of each metric family (REAL vs. DM, REAL vs. GAN, DM vs. GAN); only the DM-vs-GAN column is shown here for compactness, with the full family reported in the Appendix C, Table A2. *r* is the rank-biserial effect size. The PSD-distance row has no REAL median by construction (a reference image’s distance to its own set is identically zero and is recorded as not applicable).

Metric	Median REAL	Median DM	Median GAN	$p_{\mathrm{corr}}$ DM vs. GAN	*r* DM vs. GAN
PSD distance	n/a	0.189	0.898	<$10^{-100}$	−0.598
σ(μ) slope	0.0009	0.0045	0.0085	$5.0\times 10^{-10}$	−0.229
Short-range correlation (px)	8.45	8.14	7.97	$5.8\times 10^{-38}$	+0.204
Kurtosis (HP myocardium)	−0.43	−0.31	+1.00	<$10^{-100}$	−0.783

**Table 3:** Performance of nnU-Net on the fixed test set as a function of the number of exclusively real-world training cases. Statistical significance markers are shown for DSC and HD95 only, based on paired two-sided Wilcoxon signed-rank tests versus the 1230-case real-only baseline. Significance codes: * p<0.05, ** p<0.01, *** p<0.001.

Train	Test							
	Dice		Precision		Recall		HD95 (mm)	
Real	Myo	Scar	Myo	Scar	Myo	Scar	Myo	Scar
1230	0.8937	0.2910	0.8874	0.3974	0.9029	0.4332	2.3458	21.1866
100	0.8835 ***	0.1731 ***	0.8626	0.4564	0.9091	0.1730	2.7140 **	29.5233 ***
200	0.8870 ***	0.2223 ***	0.8650	0.4472	0.9136	0.2580	2.6515 *	23.7040 **
300	0.8881 ***	0.2315 **	0.8753	0.3627	0.9048	0.3548	2.4838	23.5770
400	0.8922	0.2540 ***	0.8774	0.4305	0.9104	0.3295	2.3197	24.4720 **
500	0.8910 *	0.2815	0.8773	0.3817	0.9080	0.4090	2.4131	20.4522
600	0.8920	0.2951 **	0.8790	0.4691	0.9086	0.3492	2.6498	24.9378

**Table 4:** Diffusion augmentation with 100 real cases. †, ††, and ††† denote statistically significant improvement vs. real (same real-case setting, Syn = 0) at Holm-corrected p<0.05, p<0.01, and p<0.001, respectively. Symbols are shown only when the significant difference is in the direction of improvement. For HD95, improvement means lower values.

Train		Test							
		Dice		Precision		Recall		HD95 (mm)	
Real	Syn	Myo	Scar	Myo	Scar	Myo	Scar	Myo	Scar
100	0	0.8835	0.1731	0.8626	0.4564	0.9091	0.1730	2.7140	29.5233
100	100	0.8842	0.2204 ^†††^	0.8743 ^†††^	0.3974	0.8979	0.2771 ^†††^	2.7952	25.1773 ^†^
100	200	0.8843	0.2529 ^†††^	0.8597	0.4063	0.9135 ^††^	0.3331 ^†††^	2.8985	21.9224 ^†††^
100	300	0.8827	0.2714 ^†††^	0.8733 ^†††^	0.4183	0.8956	0.3625 ^†††^	2.7383	23.4964 ^†††^
100	400	0.8803	0.2696 ^†††^	0.8642	0.3814	0.9012	0.3884 ^†††^	2.6860	22.4639 ^††^
100	500	0.8795	0.2377 ^†††^	0.8556	0.3414	0.9080	0.3664 ^†††^	2.9147	24.5513 ^†††^

**Table 5:** Diffusion augmentation with 300 real cases. †, ^††^, and ^†††^ denote statistically significant improvement vs. real (same real-case setting, Syn = 0) at Holm-corrected p<0.05, p<0.01, and p<0.001, respectively. Symbols are shown only when the significant difference is in the direction of improvement. For HD95, improvement means lower values.

Train		Test							
		Dice		Precision		Recall		HD95 (mm)	
Real	Syn	Myo	Scar	Myo	Scar	Myo	Scar	Myo	Scar
300	0	0.8881	0.2315	0.8753	0.3627	0.9048	0.3548	2.4838	23.5770
300	100	0.8848	0.2256	0.8798	0.4406	0.8926	0.2461	2.5246	26.4276
300	200	0.8803	0.2340	0.8602	0.3611	0.9072 ^†^	0.3431	2.6869	22.6182
300	300	0.8821	0.2353	0.8604	0.3664	0.9088 ^†††^	0.3243	2.7058	24.3353
300	400	0.8798	0.2515	0.8502	0.4241 ^†^	0.9152 ^†††^	0.3265	2.7648	22.2092
300	500	0.8844	0.2545	0.8820 ^††^	0.4307	0.8899	0.3022	2.5341	25.6208

**Table 6:** Diffusion augmentation with 600 real cases. ^††^ denotes statistically significant improvement vs. real (same real-case setting, Syn = 0) at Holm-corrected p<0.01. Symbols are shown only when the significant difference is in the direction of improvement. For HD95, improvement means lower values.

Train		Test							
		Dice		Precision		Recall		HD95 (mm)	
Real	Syn	Myo	Scar	Myo	Scar	Myo	Scar	Myo	Scar
600	0	0.8920	0.2951	0.8790	0.4691	0.9086	0.3492	2.6498	24.9378
600	100	0.8817	0.2193	0.8754	0.3803	0.8920	0.2732	2.8413	26.1073
600	200	0.8844	0.2342	0.8866 ^††^	0.3740	0.8858	0.3253	2.5407	23.6203
600	300	0.8837	0.2378	0.8717	0.3877	0.8994	0.3282	2.6955	23.2557
600	400	0.8794	0.2400	0.8619	0.4172	0.9017	0.3024	2.9215	25.7965
600	500	0.8819	0.2596	0.8590	0.4548	0.9095	0.3012	2.6778	26.8021

**Table 7:** GAN-based augmentation with 100 real cases. †, ^††^, and ^†††^ denote statistically significant improvement vs. real (same real-case setting, Syn = 0) at Holm-corrected p<0.05, p<0.01, and p<0.001, respectively; *** denotes statistically significant improvement vs. the corresponding diffusion model at Holm-corrected p<0.001. Symbols are shown only when the significant difference is in the direction of improvement. For HD95, improvement means lower values.

Train		Test							
		Dice		Precision		Recall		HD95 (mm)	
Real	Syn	Myo	Scar	Myo	Scar	Myo	Scar	Myo	Scar
100	0	0.8835	0.1731	0.8626	0.4564	0.9091	0.1730	2.7140	29.5233
100	100	0.8818	0.1812	0.4374	0.4374	0.8976	0.1930	2.7155	30.6907
100	200	0.8828	0.1923 ^†††^	0.8717 ^†††,^***	0.3557	0.8978	0.2569 ^†††^	2.4775	25.1972 ^†^
100	300	0.8822	0.1837 ^†^	0.8842 ^†††,^***	0.3892	0.8833	0.2152 ^†^	2.6395	26.6431
100	400	0.8811	0.1998 ^†††^	0.8741 ^†††,^***	0.3390	0.8919	0.2696 ^†††^	2.5535	25.6495
100	500	0.8797	0.1946	0.8692 ^††,^***	0.4001	0.8944	0.2114 ^†^	2.9296	28.6162

**Table 8:** GAN-based augmentation with 300 real cases. † and ^†††^ denote statistically significant improvement vs. real (same real-case setting, Syn = 0) at Holm-corrected p<0.05 and p<0.001, respectively; *** denotes statistically significant improvement vs. the corresponding diffusion model at Holm-corrected p<0.001. Symbols are shown only when the significant difference is in the direction of improvement. For HD95, improvement means lower values.

Train		Test							
		Dice		Precision		Recall		HD95 (mm)	
Real	Syn	Myo	Scar	Myo	Scar	Myo	Scar	Myo	Scar
300	0	0.8881	0.2315	0.8753	0.3627	0.9048	0.3548	2.4838	23.5770
300	100	0.8818	0.1867	0.8795	0.3821	0.8874	0.2158	2.6557	28.3713
300	200	0.8798	0.1669	0.8770 ***	0.3995	0.8866	0.1779	2.7935	27.1416
300	300	0.8804	0.1640	0.8586	0.4075	0.9077 ^†^	0.1737	2.6072	30.3859
300	400	0.8784	0.1976	0.8863 ^†††,^***	0.4370 ^†^	0.8749	0.2172	2.8563	26.2450
300	500	0.8768	0.1769	0.8779	0.3578	0.8806	0.2177	2.7861	28.9029

**Table 9:** GAN-based augmentation with 600 real cases. † denotes statistically significant improvement vs. real (same real-case setting, Syn = 0) at Holm-corrected p<0.05; *, **, and *** denote statistically significant improvement vs. the corresponding diffusion model at Holm-corrected p<0.05, p<0.01, and p<0.001, respectively. Symbols are shown only when the significant difference is in the direction of improvement. For HD95, improvement means lower values.

Train		Test							
		Dice		Precision		Recall		HD95 (mm)	
Real	Syn	Myo	Scar	Myo	Scar	Myo	Scar	Myo	Scar
600	0	0.8920	0.2951	0.8790	0.4691	0.9086	0.3492	2.6498	24.9378
600	100	0.8761	0.1621	0.8711	0.4882 *	0.8860	0.1523	2.8454	31.6734
600	200	0.8779	0.1540	0.8747	0.5822 ^†,^*	0.8857	0.1295	2.6350	32.3829
600	300	0.8776	0.1595	0.8828 ***	0.4730	0.8773	0.1526	2.7450	30.6468
600	400	0.8776	0.1450	0.8667 **	0.5149	0.8935	0.1249	3.1429	30.4567
600	500	0.8787	0.1460	0.8780 ***	0.5279 *	0.8839	0.1260	2.7608	30.5198

**Table 10:** Training stability of the diffusion model in the $N_{\mathrm{real}}=100$ regime, evaluated over 5 independent runs (reported as mean ± standard deviation of the Dice Similarity Coefficient, DSC). CV = coefficient of variation (%). $\Delta_{\mathrm{orig}}$ = difference between the original run and the mean across random seeds. Syn corresponds to the number of synthetic cases.

Train		Myocardium			Scar		
Real	Syn	Mean ± Std	CV%	$\Delta_{\mathrm{orig}}$	Mean ± Std	CV%	$\Delta_{\mathrm{orig}}$
100	100	0.8846 ± 0.0005	0.1	−0.0004	0.2362 ± 0.0156	6.6	−0.0158
100	200	0.8838 ± 0.0004	0.1	+0.0004	0.2465 ± 0.0061	2.5	+0.0064
100	300	0.8836 ± 0.0027	0.3	−0.0009	0.2664 ± 0.0048	1.8	+0.0050
100	400	0.8787 ± 0.0024	0.3	+0.0016	0.2427 ± 0.0280	11.5	+0.0269
100	500	0.8814 ± 0.0018	0.2	−0.0019	0.2526 ± 0.0145	5.8	−0.0149

## Data Availability

Access to a subset of the data is subject to restrictions. Proprietary datasets were provided by an industrial partner under a confidentiality agreement and cannot be made publicly available. Synthetic data generated during the course of this study are available from the corresponding author upon reasonable request.

## Appendix A. Implementation

All experiments were executed on a single NVIDIA 2090 TI GPU. To ensure deterministic reproducibility, the random seed was set to 42 for the random, NumPy, and torch libraries, including CUDA operations, and deterministic behaviour in CuDNN was enabled. For training the diffusion model, optimisation was performed with Adam using a constant learning rate of $1\times 10^{-5}$. The total computational budget for training the diffusion model amounted to 6.78 GPU-h.

Computational runtime. To complement the empirical evaluation of the complete processing pipeline, we report the computational time required for both diffusion-model-based image synthesis and nnU-Net-based segmentation inference. The diffusion model required, on average, 2.8561 s per synthesised image. By comparison, nnU-Net inference on the held-out test set comprising 120 patients required 2.7490±0.1151 s per patient (median: 2.7396 s/patient), corresponding to 0.2323±0.0226 s per slice when computed as a simple case-wise mean, or 0.2304 s per slice when calculated as a global, slice-count-weighted average.

Weighted-loss configuration. The final class-weight configuration used for the spatially weighted diffusion loss was $w_{\mathrm{bg}}=0.2$, $w_{\mathrm{myo}}=1.5$, and $w_{\mathrm{scar}}=3.0$. These weights were selected empirically through hyperparameter optimisation on a held-out validation set and were used to increase the contribution of myocardium and scar pixels during diffusion-model training.

Code and reproducibility resources. To enhance reproducibility and methodological transparency, we have established a public repository to host the full implementation, including training and sampling scripts, as well as detailed usage instructions: https://github.com/sofiamribasf/lge-dm-augmentation-segmentation.git (accessed on 24 April 2026). At the time of manuscript submission, the repository contains a placeholder README file and will be comprehensively populated upon publication.

**Table A1:** Summary of the patient-level data partitioning used for generative-model development and downstream segmentation experiments.

Component	Patients	Purpose/Partitioning Note
Full LGE-CMR cohort	1509	Source cohort employed for dataset construction (approximately 14,000 short-axis slices).
Generative model set	600	These data were utilised for training both the mask-conditioned diffusion model and the GAN-based baseline. The corresponding patients were excluded from the predefined nnU-Net validation and test cohorts.
nnU-Net real-data pool	1230	Employed both for training the real-only reference model and as the source dataset for the controlled segmentation regimes comprising 100, 300, and 600 real training instances. This pool may partially overlap with the dataset used for generative-model development.
Validation set	120	A fixed, held-out validation cohort comprising 120 cases, used exclusively for model selection and early stopping in the segmentation experiments. No patient from the generative-model development dataset was included in this set.
Test set	120	A fixed, held-out test cohort comprising 120 cases, used exclusively for the final segmentation performance assessment across all training configurations. No patients from the generative-model development dataset were included.

The only patient-level separation enforced between the generative-model development set and the downstream segmentation experiments applied to the fixed validation and test sets. Overlap with the real-data training pool used for nnU-Net training was allowed.

## Appendix C. Noise-Fidelity Analysis: Supplementary Methods, Table, and Figures

This appendix provides the methodological specification of the noise-fidelity analysis reported in Section 3.3 of the main text, the complete pairwise test statistics for all four noise-fidelity metrics (Table A2), and five supporting figures (Figure A2, Figure A3, Figure A4, Figure A5, Figure A6). Exact hyperparameter values not reproduced here are provided in the public implementation repository referenced in Appendix A.

### Appendix C.1. Per-Slice Pairing as the Statistical Unit

All cross-set noise-fidelity comparisons were performed using paired Wilcoxon signed-rank tests on per-slice triples (REAL[k],DM[k],GAN[k]) sharing the same ground-truth segmentation mask at slice key *k*. Per-slice rather than per-patient pairing is the appropriate statistical unit in this analysis because the diffusion and GAN generators were conditioned slice-wise, with no enforced volumetric coherence between adjacent slices: each generated slice is an independent sample from its generator conditioned on a single mask, so aggregating to per-patient medians would discard information and artificially reduce power.

To guard against the residual within-patient dependency introduced by multiple slices per subject, a within-patient resampling sensitivity analysis was also performed (Appendix C.5; Figure A6). At the cohort’s acquired inter-slice spacing (nominally 10 mm, effective range 8–15 mm after accounting for slice thickness, with 7–16 short-axis slices covering each heart from base to apex), scanner noise realisations are uncorrelated across slices by construction, fibrotic scar rarely spans adjacent slices, and the extra-myocardial tissue region that dominates the per-image noise-standard-deviation estimate varies substantially between slices. The per-patient resampling analysis, therefore, serves as a strict robustness check rather than one expected to be load-bearing, and indeed all four headline DM-vs-GAN contrasts survive at ≥94.8% robustness, with three of the four at ≥99.9% (Table A2); the narrowest margin is the σ(μ)-slope contrast at 94.8%.

### Appendix C.2. Per-Image Noise Standard Deviation via Wavelet MAD

The per-image noise standard deviation $\hat{\sigma }$ used to construct the SNR distribution of Figure A2 was estimated via the wavelet median-absolute-deviation (MAD) estimator of Donoho and Johnstone [45], following the medical-imaging adaptation of Coupé et al. [46]. Concretely, each image was decomposed at one scale using the Daubechies-4 orthonormal wavelet, and $\hat{\sigma }$ was computed from the diagonal (HH) detail coefficients as

(A1) $$\hat{\sigma }\;=\;\frac{\mathrm{median}\left(|c_{HH}|\right)}{0.6745},$$

where the 0.6745 constant is the Gaussian consistency factor. The estimator was applied exclusively to the extra-myocardial tissue region (i.e., tissue pixels outside both the myocardium and scar labels). For the paired REAL–DM–GAN comparisons that follow, the tissue mask was computed once per slice from the REAL image and reused for the DM and GAN $\hat{\sigma }$ estimates, so that all three per-image $\hat{\sigma }$ values at a given slice key were computed on identical pixel support; this ensures that any cross-method difference in $\hat{\sigma }$ reflects the image content rather than a method-dependent choice of field-of-view threshold. To exclude wavelet-support contamination at the mask boundary, the downsampled mask (at the resolution of the detail sub-band) was further eroded by one detail-scale pixel before indexing the coefficients; detail coefficients at pixels whose 2-px filter support crossed the mask boundary are therefore not included in the median.

Wavelet MAD was selected in preference to the classical air-region-of-interest Rayleigh fit for two reasons. First, the 128×128 cardiac crops used throughout this study do not contain extracorporeal air, so an air-ROI estimator is unavailable. Second, the parallel-imaging reconstructions used for this cohort produce spatially non-stationary noise that biases air-ROI Rayleigh fits even when extracorporeal air is available, whereas wavelet MAD is robust to this non-stationarity because its localised support adapts to the tissue region being analysed.

### Appendix C.3. Signal-Dependent Variance σ(μ) and the Windowed-Statistics Support Criterion

For each image, signal-dependent variance was characterised by the local relationship between mean intensity and standard deviation within the myocardial ROI. A sliding square window (edge length 7 px) was stepped across the ROI; at each window position for which valid mask coverage exceeded 75%, a paired estimate $\left(\mu_{w},\sigma_{w}\right)$ of the local mean and local standard deviation was recorded. Window positions straddling the mask boundary with less than 75% coverage were rejected to avoid edge-induced variance inflation.

Two estimators were used at each window, on different fields. The local mean $\mu_{w}$ is the arithmetic mean of the raw in-mask pixel values within the window: this is the quantity that serves as the “local signal level” on the σ(μ) abscissa. The local standard deviation $\sigma_{w}$ is the root-mean-square of a detrended residual at the window, specifically $\sigma_{w}^{2}={\langle {\left(I-G_{\sigma_{d}}*I\right)}^{2}\rangle }_{w}$, where $G_{\sigma_{d}}$ is a Gaussian kernel of standard deviation $\sigma_{d}=2$ px and the angle brackets denote the within-window in-mask average. This construction separates the two estimators: $\mu_{w}$ tracks signal gradients on the window scale (preserved because the detrending kernel has narrower support than the window), while $\sigma_{w}$ suppresses those gradients and retains the noise component, so that the measured slope of σ(μ) reflects signal-dependent noise rather than a mixture of noise variance and residual anatomical structure. With $\sigma_{d}=2$ px and a 7 px window, window-scale structure is captured in μ while pixel-scale and near-pixel-scale noise is captured in σ; a legacy path with $\sigma_{d}=0$ (which recovers the classical within-window centred-variance estimator $\sigma_{w}^{2}=\mathrm{Var}{\left(I\right)}_{w}$, i.e., both μ and σ from the raw image) is retained in the public implementation for comparison.

The per-window pairs $\left(\mu_{w},\sigma_{w}\right)$ were binned along the μ-axis over the full intensity range [0,1], and the per-bin mean $\bar{\sigma }\left(\mu \right)$ curves were aggregated across slices to produce Figure 4b in the main text. The per-image σ(μ) slope reported in Table 2 of the main text and in Table A2 was computed by linear regression of $\sigma_{w}^{2}$ on $\mu_{w}$ over the $\left(\mu_{w},\sigma_{w}\right)$ pairs from a given slice.

A slice was retained for the slope analysis only if it met a windowed-statistics support criterion: at least 20 valid window positions had to be available within the healthy-myocardium ROI (myocardium with scar-adjacent regions excluded by a 2-px dilation of the scar mask) for the slope to be stably estimated. Slices with small myocardial areas (typically basal or apical slices with limited muscle visible) and slices where the scar exclusion reduced the healthy-myocardium ROI to fewer than the required window count were excluded. Of the 5591 slice triples available, 1036 met this criterion and were included in the σ(μ)-slope comparisons; the remaining triples contributed to the σ(μ)-curve visualisation shown in Figure 4b of the main text but not to the scalar slope test. Exact window size, bin count, and support thresholds are specified in the public implementation repository.

### Appendix C.4. Short-Range Spatial Correlation Metric

Short-range spatial correlation was quantified as the integral of the normalised radial autocorrelation function of the high-pass-filtered myocardial ROI over lags [0,10] px. For each slice and image set, the procedure was as follows:

1. Apply a high-pass filter to the myocardial ROI to isolate the noise component from the slowly varying anatomical signal (implementation: Gaussian high-pass by subtracting a Gaussian-smoothed version of the ROI, with a cut-off chosen to suppress structure at scales larger than the expected noise correlation length).
2. Compute the 2D autocorrelation of the high-pass residual by FFT, restricted to pixels within the myocardial mask.
3. Convert this to a radial autocorrelation by averaging over angular bins at fixed radii.
4. Normalise it so that the zero-lag value equals unity.
5. Integrate the normalised radial autocorrelation over the lag range [0,10] px to produce a single scalar per slice and per image set.

Larger values of this scalar indicate greater spatial correlation of noise at short lags, which is the scale range most strongly influenced by *k*-space filtering, parallel-imaging reconstruction, and receive-coil geometry.

A slice triple was excluded from the comparison if the myocardial ROI in any of the three image sets (REAL, DM, GAN) did not support stable estimation of the autocorrelation over the [0,10] px lag range with sufficient mask overlap—in practice, this occurred in cases where the myocardial ring was thin enough that the 10-px lag exceeded the typical chord length within the mask. Of the 5591 available slice triples, 180 were excluded by this criterion, leaving n=5411 triples for the comparison reported in the main text, Table 2, and in the Appendix C, Table A2.

### Appendix C.5. Within-Patient Resampling Sensitivity Analysis

The within-patient resampling procedure summarised in the main-text robustness percentages and visualised in Figure A6 was performed as follows. In each of 1000 independent iterations, one slice was drawn uniformly at random from each patient, yielding n≈639 slices per iteration. On this reduced set, the three paired Wilcoxon signed-rank tests (REAL vs. DM, REAL vs. GAN, DM vs. GAN) were rerun with Bonferroni correction across the three pairwise contrasts within each metric family. Per-iteration rank-biserial effect sizes and Bonferroni-corrected *p*-values were recorded.

The robustness fraction reported alongside each effect size in Table A2 is the proportion of the 1000 iterations in which the comparison remained statistically significant at $p_{\mathrm{corr}}<0.05$. Because each resampled dataset is drawn independently and contains exactly one slice per patient, the resampling distribution directly addresses the concern that multiple slices per patient could inflate the effective sample size of the per-slice paired tests. A finding with a robustness fraction close to 1 is insensitive to this source of pseudoreplication; a finding with a robustness fraction close to 0 is driven by within-patient dependency and should not be regarded as generalising to a per-patient inference target.

### Appendix C.6. Radial Noise PSD and the Mask-Autocorrelation Bias Correction

The radial power spectral density reported in Figure 4a of the main text and quantified as PSD distance to REAL in Table 2 row 1 of the main text is computed from the noise component of each image rather than from the raw intensity field, and is corrected for the spectral bias introduced by the finite-support myocardial mask. The procedure for a single slice and image set is as follows:

1. Apply a Gaussian high-pass pre-filter to the full image: $H=I-G_{\sigma_{\mathrm{hp}}}*I$ with $\sigma_{\mathrm{hp}}=6$ px. This suppresses tissue-scale anatomical structure; the noise band of interest lies at higher spatial frequencies than the pre-filter cut-off.
2. Restrict the high-pass image to the myocardial mask *M* and subtract the in-mask mean to remove any residual DC offset inside the ROI: $\tilde{H}=\left(H-{\langle H\rangle }_{M}\right)⊙M$. This pre-subtraction is essential because a nonzero mean inside the mask creates a step discontinuity at the mask boundary and injects a mask-shaped low-frequency contribution into the subsequent FFT.
3. Compute the biased autocorrelation of the zero-filled masked image by FFT: $A_{\mathrm{img}}=\mathrm{IFFT}(|\mathrm{FFT}\left(\tilde{H}\right){|}^{2})$.
4. Compute the autocorrelation of the mask itself: $A_{\mathrm{mask}}={\mathrm{IFFT}(|\mathrm{FFT}\left(M\right)|}^{2})$. For each lag, this quantity counts the number of in-mask pixel pairs separated by that lag.
5. Divide to obtain an unbiased estimate of the noise autocorrelation, $A=A_{\mathrm{img}}/\max\left(A_{\mathrm{mask}},1\right)$. This step removes the dependence of the spectrum on the mask shape; without it, irregular or small ROIs produce spectra dominated by the mask’s own Fourier transform.
6. Apply a separable 2-D Tukey window (α=0.5) to the centred autocorrelation, then compute the forward FFT to obtain a smoothed, positive-valued PSD estimate.
7. Radially average the result over 32 frequency bins in [0,0.5] cycles/pixel to yield the per-image radial PSD curve.

The per-image PSD distance to REAL is the mean-squared difference between the base-10 logarithms of the per-image PSD and the reference REAL PSD for the same slice, with a scale-aware floor of $10^{-6}\cdot \max\left(\mathrm{PSD}\right)$ to prevent a single near-zero bin from dominating the mean. Under this metric, the PSD distance of a REAL image to its own reference is identically zero, so REAL-vs-DM and REAL-vs-GAN rows of the PSD-distance family are reported as not applicable in Table A2; the inter-generator contrast (DM vs. GAN) is the only well-defined comparison for this metric.

The Gaussian high-pass pre-filter leaves a visible roll-off in the lowest frequency bins of all three PSD curves in Figure 4a of the main text, at frequencies below approximately 0.03 cycles/px (≈$1/(2\pi \sigma_{\mathrm{hp}})$). Because the same pre-filter is applied to REAL, DM, and GAN, this feature does not bias the cross-method comparison; it should, however, be read as a signature of the pre-filter rather than as a scanner-specific spectral peak.

**Table A2:** Full pairwise test statistics for the four noise-fidelity metrics. All tests are paired Wilcoxon signed-rank tests on per-slice triples sharing the ground-truth mask; $p_{\mathrm{corr}}$ is Bonferroni-corrected across the three pairwise contrasts within each metric family. The rank-biserial correlation *r* is the primary effect-size measure. The robustness column reports the percentage of 1000 within-patient resampling iterations in which the comparison remained significant at $p_{\mathrm{corr}}<0.05$. PSD-distance comparisons involving REAL are not applicable because REAL is the reference arm for this metric.

Metric Family	Comparison	*n*	Med. A	Med. B	*r*	$p_{\mathrm{corr}}$	Robust. (%)
PSD distance to REAL	REAL vs. DM	–	n/a	0.189	n/a	n/a	n/a
	REAL vs. GAN	–	n/a	0.898	n/a	n/a	n/a
	DM vs. GAN	5537	0.189	0.898	−0.598	<$10^{-100}$	100.0
σ(μ) slope	REAL vs. DM	1036	0.0009	0.0045	−0.282	$1.1\times 10^{-14}$	99.8
	REAL vs. GAN	1036	0.0009	0.0085	−0.511	$1.6\times 10^{-45}$	100.0
	DM vs. GAN	1036	0.0045	0.0085	−0.229	$5.0\times 10^{-10}$	94.8
Short-range correlation (px)	REAL vs. DM	5411	8.45	8.14	+0.401	$9.1\times 10^{-144}$	100.0
	REAL vs. GAN	5411	8.45	7.97	+0.530	$8.7\times 10^{-250}$	100.0
	DM vs. GAN	5411	8.14	7.97	+0.204	$5.8\times 10^{-38}$	99.9
Kurtosis (HP myocardium)	REAL vs. DM	5586	−0.43	−0.31	−0.214	$2.8\times 10^{-43}$	99.1
	REAL vs. GAN	5586	−0.43	+1.00	−0.855	<$10^{-100}$	100.0
	DM vs. GAN	5586	−0.31	+1.00	−0.783	<$10^{-100}$	100.0

## Appendix D. Additional Statistical Analysis Details

**Table A3:** Segmentation performance (mean [95% bootstrap CI]) for Myocardium using models trained with real data only. Statistical comparison versus the baseline model trained with 1230 real cases was performed using the Wilcoxon signed-rank test (two-sided, paired at the patient level). Stat. columns report the test statistic *W*, the paired *p*-value, and the rank-biserial correlation *r* for DSC, HD95, precision, and recall. HD95 is reported in mm. Significance codes, where present, are: * p<0.05, ** p<0.01, and *** p<0.001.

Real	DSC	Stat.	HD95	Stat.	Precision	Stat.	Recall	Stat.
1230 (baseline)	0.894 [0.885–0.902]	—	2.346 [2.089–2.641]	—	0.887 [0.876–0.898]	—	0.903 [0.893–0.912]	—
100	0.884 [0.872–0.893]	*W* = 1152 <0.001 *** (0.84)	2.714 [2.366–3.106]	*W* = 78 0.008 ** (0.99)	0.863 [0.849–0.875]	*W* = 371 <0.001 *** (0.95)	0.909 [0.896–0.920]	*W* = 1778 <0.001 *** (0.76)
200	0.887 [0.876–0.896]	*W* = 1878 <0.001 *** (0.74)	2.652 [2.316–3.029]	*W* = 75 0.018 * (0.99)	0.865 [0.851–0.877]	*W* = 155 <0.001 *** (0.98)	0.914 [0.903–0.923]	*W* = 1065 <0.001 *** (0.85)
300	0.888 [0.878–0.897]	*W* = 1780 <0.001 *** (0.75)	2.484 [2.177–2.832]	*W* = 57 0.356 (0.99)	0.875 [0.862–0.887]	*W* = 816 <0.001 *** (0.89)	0.905 [0.893–0.915]	*W* = 2603 0.007 ** (0.64)
400	0.892 [0.883–0.900]	*W* = 3269 0.344 (0.55)	2.320 [2.045–2.638]	*W* = 55 0.776 (0.99)	0.877 [0.866–0.888]	*W* = 1250 <0.001 *** (0.83)	0.910 [0.900–0.920]	*W* = 1383 <0.001 *** (0.81)
500	0.891 [0.882–0.899]	*W* = 2793 0.028 * (0.62)	2.413 [2.125–2.742]	*W* = 43.5 0.572 (0.99)	0.877 [0.866–0.887]	*W* = 1148 <0.001 *** (0.84)	0.908 [0.897–0.918]	*W* = 1861 <0.001 *** (0.74)
600	0.892 [0.883–0.900]	*W* = 3382 0.516 (0.53)	2.650 [2.258–3.090]	*W* = 26 0.096 (1.00)	0.879 [0.867–0.890]	*W* = 1826 <0.001 *** (0.75)	0.909 [0.899–0.918]	*W* = 1985 <0.001 *** (0.73)

**Table A4:** Segmentation performance (mean [95% bootstrap CI]) for Scar using models trained with real data only. Statistical comparison versus the baseline model trained with 1230 real cases was performed using the Wilcoxon signed-rank test (two-sided, paired at the patient level). Stat. columns report the test statistic *W*, the paired *p*-value, and the rank-biserial correlation *r* for DSC, HD95, precision, and recall. HD95 is reported in mm. Significance codes, where present, are: * p<0.05, ** p<0.01, and *** p<0.001.

Real	DSC	Stat.	HD95	Stat.	Precision	Stat.	Recall	Stat.
1230 (baseline)	0.291 [0.228–0.356]	—	21.187 [17.053–25.740]	—	0.397 [0.317–0.477]	—	0.433 [0.361–0.505]	—
100	0.173 [0.126–0.223]	*W* = 34 <0.001 *** (0.98)	29.523 [23.880–35.480]	*W* = 122 <0.001 *** (0.85)	0.456 [0.357–0.556]	*W* = 288 0.155 (0.81)	0.173 [0.127–0.222]	*W* = 21 <0.001 *** (0.98)
200	0.222 [0.166–0.281]	*W* = 78 <0.001 *** (0.97)	23.704 [19.033–28.796]	*W* = 250.5 0.004 ** (0.75)	0.447 [0.355–0.543]	*W* = 245 0.016 * (0.88)	0.258 [0.197–0.319]	*W* = 56 <0.001 *** (0.96)
300	0.232 [0.177–0.286]	*W* = 330 0.008 ** (0.88)	23.577 [19.208–28.363]	*W* = 433 0.340 (0.65)	0.363 [0.287–0.438]	*W* = 439 0.267 (0.84)	0.355 [0.285–0.426]	*W* = 310 0.007 ** (0.77)
400	0.254 [0.194–0.316]	*W* = 238 <0.001 *** (0.91)	24.472 [19.870–29.234]	*W* = 302 0.009 ** (0.74)	0.430 [0.347–0.516]	*W* = 327 0.020 * (0.86)	0.330 [0.263–0.396]	*W* = 83 <0.001 *** (0.94)
500	0.281 [0.221–0.345]	*W* = 519 0.253 (0.82)	20.452 [16.481–24.946]	*W* = 545 0.846 (0.52)	0.382 [0.307–0.458]	*W* = 530 0.909 (0.80)	0.409 [0.340–0.476]	*W* = 469 0.153 (0.65)
600	0.295 [0.229–0.362]	*W* = 238 0.001 *** (0.90)	24.938 [19.671–30.588]	*W* = 369 0.061 (0.69)	0.469 [0.377–0.560]	*W* = 399 0.263 (0.82)	0.349 [0.278–0.420]	*W* = 115 <0.001 *** (0.91)

**Table A5:** Segmentation performance (mean [95% bootstrap CI]) for Myocardium, comparing the real-only baseline and diffusion-based augmentation in the $N_{\mathrm{real}}=100$ regime. Real = number of real training cases; Syn = synthetic cases added. Statistical comparison versus the corresponding real-only baseline (same number of real cases, same test set) is reported for DSC, HD95, precision, and recall using the Wilcoxon signed-rank test (two-sided, paired at the patient level), with Holm-corrected *p*-values within the three-way comparison framework. Stat. columns report the test statistic *W*, corrected *p*-value ($p_{c}$), and the rank-biserial correlation *r*. HD95 is reported in mm. Significance codes, where present, are: * p<0.05, ** p<0.01, and *** p<0.001.

Real	Syn	DSC	Stat.	HD95	Stat.	Precision	Stat.	Recall	Stat.
100	0	0.883 [0.872–0.893]	—	2.714 [2.366–3.106]	—	0.863 [0.849–0.875]	—	0.909 [0.896–0.920]	—
100	100	0.884 [0.872–0.894]	*W* = 3120 0.364 (0.57)	2.795 [2.395–3.255]	*W* = 144 1.000 (0.98)	0.874 [0.861–0.886]	*W* = 1387 <0.001 *** (0.81)	0.898 [0.885–0.909]	*W* = 1179 <0.001 *** (0.84)
100	200	0.884 [0.874–0.893]	*W* = 3301 0.494 (0.55)	2.898 [2.456–3.397]	*W* = 205 0.787 (0.97)	0.860 [0.847–0.872]	*W* = 3061 0.136 (0.58)	0.913 [0.902–0.923]	*W* = 2515 0.008 ** (0.65)
100	300	0.883 [0.872–0.892]	*W* = 3378 0.692 (0.54)	2.738 [2.389–3.128]	*W* = 151.5 1.000 (0.98)	0.873 [0.861–0.885]	*W* = 1583 <0.001 *** (0.78)	0.896 [0.883–0.906]	*W* = 978 <0.001 *** (0.86)
100	400	0.880 [0.868–0.890]	*W* = 2820 0.068 (0.61)	2.686 [2.306–3.133]	*W* = 161.5 0.866 (0.98)	0.864 [0.853–0.875]	*W* = 3502 0.737 (0.52)	0.901 [0.886–0.914]	*W* = 2208.5 <0.001 *** (0.70)
100	500	0.879 [0.868–0.889]	*W* = 2922 0.128 (0.60)	2.915 [2.516–3.366]	*W* = 188 1.000 (0.97)	0.856 [0.842–0.868]	*W* = 2626 0.009 ** (0.64)	0.908 [0.895–0.919]	*W* = 3517 0.767 (0.52)

**Table A6:** Segmentation performance (mean [95% bootstrap CI]) for Scar, comparing the real-only baseline and diffusion-based augmentation in the $N_{\mathrm{real}}=100$ regime. Real = number of real training cases; Syn = synthetic cases added. Statistical comparison versus the corresponding real-only baseline (same number of real cases, same test set) is reported for DSC, HD95, precision, and recall using the Wilcoxon signed-rank test (two-sided, paired at the patient level), with Holm-corrected *p*-values within the three-way comparison framework. Stat. columns report the test statistic *W*, corrected *p*-value ($p_{c}$), and the rank-biserial correlation *r*. HD95 is reported in mm. Significance codes, where present, are: * p<0.05, ** p<0.01, and *** p<0.001.

Real	Syn	DSC	Stat.	HD95	Stat.	Precision	Stat.	Recall	Stat.
100	0	0.173 [0.126–0.223]	—	29.523 [23.880–35.480]	—	0.456 [0.357–0.556]	—	0.173 [0.127–0.222]	—
100	100	0.220 [0.167–0.273]	*W* = 120 <0.001 *** (0.95)	25.177 [20.669–29.865]	*W* = 198 0.021 * (0.76)	0.397 [0.316–0.481]	*W* = 227 0.111 (0.87)	0.277 [0.225–0.331]	*W* = 101 <0.001 *** (0.92)
100	200	0.253 [0.195–0.312]	*W* = 60 <0.001 *** (0.97)	21.922 [17.595–26.518]	*W* = 84 <0.001 *** (0.90)	0.406 [0.323–0.489]	*W* = 245 0.054 (0.86)	0.333 [0.273–0.394]	*W* = 32 <0.001 *** (0.98)
100	300	0.271 [0.211–0.332]	*W* = 39 <0.001 *** (0.98)	23.496 [18.969–28.253]	*W* = 154 <0.001 *** (0.81)	0.418 [0.336–0.500]	*W* = 251 0.156 (0.85)	0.362 [0.302–0.421]	*W* = 27 <0.001 *** (0.98)
100	400	0.270 [0.211–0.328]	*W* = 37 <0.001 *** (0.98)	22.464 [18.424–26.750]	*W* = 162 0.003 ** (0.80)	0.381 [0.304–0.459]	*W* = 170 0.004 ** (0.90)	0.388 [0.328–0.449]	*W* = 28 <0.001 *** (0.98)
100	500	0.238 [0.184–0.294]	*W* = 106 <0.001 *** (0.96)	24.551 [20.259–29.221]	*W* = 146 <0.001 *** (0.82)	0.341 [0.270–0.416]	*W* = 182 0.012 * (0.89)	0.366 [0.304–0.429]	*W* = 82 <0.001 *** (0.94)

### Loss-Weighting Ablation in the Low-Data Diffusion Regime

To quantify the impact of the weighted-loss formulation employed for diffusion-based augmentation in the low-data regime, we conducted an additional paired ablation study in the $N_{\mathrm{real}}=100$ setting. Specifically, we compared the standard loss weighting scheme with an otherwise identical unweighted variant across all five diffusion-model (DM) synthetic data increments. Statistical inference was carried out using two-sided paired Wilcoxon signed-rank tests on per-patient performance scores, with Holm–Bonferroni correction applied across the five increment levels within each tissue × metric combination.

For the scar class, loss weighting predominantly enhanced Dice and recall relative to the unweighted variant, with statistically significant improvements in Dice at all increment levels and in recall at all but the highest augmentation level. Improvements in scar HD95 were more modest and reached statistical significance only at increments of 100 and 400, while no statistically significant gains were observed for scar precision. For the myocardium, the effects of loss weighting were mixed and of smaller practical magnitude, supporting the interpretation that the primary benefit of the weighted-loss formulation is improved management of class imbalance for scar segmentation in the low-data setting.

**Table A7:** Effect of loss weighting on segmentation performance (R100 + DM augmentation). P = with weighting, NP = without weighting. Δ = NP − P (mean difference). For HD95, a negative Δ indicates NP achieves lower (better) distances. Significance of the pairwise Wilcoxon signed-rank test (two-sided, paired at the patient level; Holm–Bonferroni corrected across the five increment levels within each tissue×metric group): * p<0.05, ** p<0.01, *** p<0.001.

Tissue		Dice			HD95 (mm)			Recall			Precision		
	Syn	P	NP	Δ	P	NP	Δ	P	NP	Δ	P	NP	Δ
Myocardium	100	0.884	0.874	−0.011 ***	2.80	3.30	+0.51	0.898	0.899	+0.001	0.874	0.854	−0.021 ***
	200	0.884	0.882	−0.002 *	2.90	2.54	−0.36	0.913	0.903	−0.011 ***	0.860	0.865	+0.006 *
	300	0.883	0.883	+0.000	2.74	2.65	−0.09	0.896	0.889	−0.007 ***	0.873	0.880	+0.007 ***
	400	0.880	0.884	+0.004 **	2.69	2.48	−0.20	0.901	0.900	−0.002 *	0.864	0.872	+0.008 ***
	500	0.879	0.881	+0.002	2.91	2.70	−0.21	0.908	0.896	−0.011 ***	0.856	0.872	+0.016 ***
Scar	100	0.220	0.166	−0.055 ***	25.18	30.89	+5.72 *	0.277	0.149	−0.128 ***	0.397	0.470	+0.073
	200	0.253	0.212	−0.041 ***	21.92	25.28	+3.36	0.333	0.223	−0.110 ***	0.406	0.440	+0.033
	300	0.271	0.214	−0.058 ***	23.50	24.18	+0.68	0.362	0.260	−0.103 ***	0.418	0.378	−0.041
	400	0.270	0.234	−0.035 **	22.46	25.82	+3.35 *	0.388	0.313	−0.076 **	0.381	0.350	−0.031
	500	0.238	0.191	−0.047 ***	24.55	28.20	+3.65	0.366	0.253	−0.113 ***	0.341	0.347	+0.005

**Table A8:** Segmentation performance (mean [95% bootstrap CI]) for Myocardium, comparing the real-only baseline and diffusion-based augmentation in the $N_{\mathrm{real}}=300$ regime. Real = number of real training cases; Syn = synthetic cases added. Statistical comparison versus the corresponding real-only baseline (same number of real cases, same test set) is reported for DSC, HD95, precision, and recall using the Wilcoxon signed-rank test (two-sided, paired at the patient level), with Holm-corrected *p*-values within the three-way comparison framework. Stat. columns report the test statistic *W*, corrected *p*-value ($p_{c}$), and the rank-biserial correlation *r*. HD95 is reported in mm. Significance codes, where present, are: * p<0.05, ** p<0.01, and *** p<0.001.

Real	Syn	DSC	Stat.	HD95	Stat.	Precision	Stat.	Recall	Stat.
300	0	0.888 [0.878–0.897]	—	2.484 [2.177–2.832]	—	0.875 [0.862–0.887]	—	0.905 [0.893–0.915]	—
300	100	0.885 [0.875–0.894]	*W* = 2367 0.002 ** (0.67)	2.525 [2.232–2.859]	*W* = 117 0.249 (0.98)	0.880 [0.869–0.890]	*W* = 2875 0.130 (0.60)	0.893 [0.880–0.903]	*W* = 1019 <0.001 *** (0.86)
300	200	0.880 [0.868–0.890]	*W* = 1896 <0.001 *** (0.74)	2.687 [2.263–3.230]	*W* = 120 0.782 (0.98)	0.860 [0.849–0.870]	*W* = 776 <0.001 *** (0.89)	0.907 [0.891–0.919]	*W* = 2642 0.010 * (0.64)
300	300	0.882 [0.870–0.892]	*W* = 2124 <0.001 *** (0.71)	2.706 [2.314–3.172]	*W* = 113 0.336 (0.98)	0.860 [0.848–0.871]	*W* = 1116 <0.001 *** (0.85)	0.909 [0.894–0.921]	*W* = 2123 <0.001 *** (0.71)
300	400	0.880 [0.868–0.889]	*W* = 1657 <0.001 *** (0.77)	2.765 [2.341–3.251]	*W* = 114 0.192 (0.98)	0.850 [0.837–0.862]	*W* = 262 <0.001 *** (0.96)	0.915 [0.902–0.926]	*W* = 1144 <0.001 *** (0.84)
300	500	0.884 [0.873–0.894]	*W* = 2667 0.012 * (0.63)	2.534 [2.215–2.902]	*W* = 155.5 0.421 (0.98)	0.882 [0.870–0.893]	*W* = 2474 0.006 ** (0.66)	0.890 [0.877–0.901]	*W* = 1437 <0.001 *** (0.80)

**Table A9:** Segmentation performance (mean [95% bootstrap CI]) for Scar, comparing the real-only baseline and diffusion-based augmentation in the $N_{\mathrm{real}}=300$ regime. Real = number of real training cases; Syn = synthetic cases added. Statistical comparison versus the corresponding real-only baseline (same number of real cases, same test set) is reported for DSC, HD95, precision, and recall using the Wilcoxon signed-rank test (two-sided, paired at the patient level), with Holm-corrected *p*-values within the three-way comparison framework. Stat. columns report the test statistic *W*, corrected *p*-value ($p_{c}$), and the rank-biserial correlation *r*. HD95 is reported in mm. Significance codes, where present, are: * p<0.05, ** p<0.01, and *** p<0.001.

Real	Syn	DSC	Stat.	HD95	Stat.	Precision	Stat.	Recall	Stat.
300	0	0.232 [0.177–0.286]	—	23.577 [19.208–28.363]	—	0.363 [0.287–0.438]	—	0.355 [0.285–0.426]	—
300	100	0.226 [0.171–0.283]	*W* = 222 <0.001 *** (0.91)	26.428 [20.949–32.402]	*W* = 294 0.098 (0.69)	0.441 [0.346–0.536]	*W* = 246 0.054 (0.87)	0.246 [0.192–0.300]	*W* = 141 <0.001 *** (0.89)
300	200	0.234 [0.179–0.290]	*W* = 611 0.988 (0.81)	22.618 [18.130–27.344]	*W* = 481 0.272 (0.62)	0.361 [0.283–0.439]	*W* = 531 1.000 (0.83)	0.343 [0.281–0.406]	*W* = 564 1.000 (0.57)
300	300	0.235 [0.180–0.292]	*W* = 538 0.608 (0.82)	24.335 [19.457–29.742]	*W* = 538 0.978 (0.52)	0.366 [0.286–0.448]	*W* = 478 0.843 (0.82)	0.324 [0.263–0.386]	*W* = 478 0.363 (0.64)
300	400	0.252 [0.194–0.313]	*W* = 572 0.527 (0.80)	22.209 [18.002–26.813]	*W* = 492 0.972 (0.55)	0.424 [0.338–0.509]	*W* = 287 0.018 * (0.89)	0.327 [0.263–0.390]	*W* = 491 0.227 (0.63)
300	500	0.255 [0.197–0.314]	*W* = 464 0.203 (0.82)	25.621 [20.768–30.966]	*W* = 446 0.212 (0.62)	0.431 [0.347–0.518]	*W* = 423 0.572 (0.82)	0.302 [0.242–0.363]	*W* = 375 0.045 * (0.72)

**Table A10:** Segmentation performance (mean [95% bootstrap CI]) for Myocardium, comparing the real-only baseline and diffusion-based augmentation in the $N_{\mathrm{real}}=600$ regime. Real = number of real training cases; Syn = synthetic cases added. Statistical comparison versus the corresponding real-only baseline (same number of real cases, same test set) is reported for DSC, HD95, precision, and recall using the Wilcoxon signed-rank test (two-sided, paired at the patient level), with Holm-corrected *p*-values within the three-way comparison framework. Stat. columns report the test statistic *W*, corrected *p*-value ($p_{c}$), and the rank-biserial correlation *r*. HD95 is reported in mm. Significance codes, where present, are: * p<0.05, ** p<0.01, and *** p<0.001.

Real	Syn	DSC	Stat.	HD95	Stat.	Precision	Stat.	Recall	Stat.
600	0	0.892 [0.883–0.900]	—	2.650 [2.258–3.090]	—	0.879 [0.867–0.890]	—	0.909 [0.899–0.918]	—
600	100	0.882 [0.870–0.891]	*W* = 815 <0.001 *** (0.89)	2.841 [2.430–3.322]	*W* = 144 0.207 (0.98)	0.875 [0.864–0.886]	*W* = 2853 0.042 * (0.61)	0.892 [0.878–0.904]	*W* = 1008 <0.001 *** (0.86)
600	200	0.884 [0.873–0.893]	*W* = 1378 <0.001 *** (0.81)	2.541 [2.213–2.940]	*W* = 221 0.831 (0.97)	0.887 [0.875–0.897]	*W* = 2318 0.002 ** (0.68)	0.886 [0.872–0.897]	*W* = 232 <0.001 *** (0.97)
600	300	0.884 [0.874–0.892]	*W* = 1132 <0.001 *** (0.84)	2.696 [2.328–3.116]	*W* = 153.5 0.834 (0.98)	0.872 [0.860–0.882]	*W* = 1987 <0.001 *** (0.73)	0.899 [0.887–0.910]	*W* = 1697 <0.001 *** (0.77)
600	400	0.879 [0.868–0.889]	*W* = 841 <0.001 *** (0.88)	2.921 [2.467–3.464]	*W* = 194.5 0.248 (0.97)	0.862 [0.850–0.873]	*W* = 869 <0.001 *** (0.88)	0.902 [0.888–0.913]	*W* = 2799 0.030 * (0.61)
600	500	0.882 [0.871–0.891]	*W* = 1136 <0.001 *** (0.84)	2.678 [2.354–3.043]	*W* = 245 0.738 (0.97)	0.859 [0.847–0.870]	*W* = 848 <0.001 *** (0.88)	0.909 [0.897–0.920]	*W* = 3030 0.116 (0.58)

**Table A11:** Segmentation performance (mean [95% bootstrap CI]) for Scar, comparing the real-only baseline and diffusion-based augmentation in the $N_{\mathrm{real}}=600$ regime. Real = number of real training cases; Syn = synthetic cases added. Statistical comparison versus the corresponding real-only baseline (same number of real cases, same test set) is reported for DSC, HD95, precision, and recall using the Wilcoxon signed-rank test (two-sided, paired at the patient level), with Holm-corrected *p*-values within the three-way comparison framework. Stat. columns report the test statistic *W*, corrected *p*-value ($p_{c}$), and the rank-biserial correlation *r*. HD95 is reported in mm. Significance codes, where present, are: * p<0.05, ** p<0.01, and *** p<0.001.

Real	Syn	DSC	Stat.	HD95	Stat.	Precision	Stat.	Recall	Stat.
600	0	0.295 [0.229–0.362]	—	24.938 [19.671–30.588]	—	0.469 [0.377–0.560]	—	0.349 [0.278–0.420]	—
600	100	0.219 [0.165–0.274]	*W* = 266 0.003 ** (0.88)	26.107 [21.008–31.502]	*W* = 397 0.496 (0.62)	0.380 [0.297–0.466]	*W* = 360 0.258 (0.80)	0.273 [0.216–0.331]	*W* = 266 0.005 ** (0.80)
600	200	0.234 [0.178–0.292]	*W* = 480 0.268 (0.80)	23.620 [19.231–28.366]	*W* = 482 0.879 (0.53)	0.374 [0.294–0.456]	*W* = 371 0.314 (0.82)	0.325 [0.263–0.387]	*W* = 504 0.525 (0.62)
600	300	0.238 [0.184–0.292]	*W* = 583 0.959 (0.75)	23.256 [18.897–28.062]	*W* = 467 0.569 (0.60)	0.388 [0.309–0.470]	*W* = 497 1.000 (0.77)	0.328 [0.271–0.385]	*W* = 557 0.941 (0.58)
600	400	0.240 [0.182–0.302]	*W* = 335 0.039 * (0.86)	25.797 [20.686–31.368]	*W* = 444 0.925 (0.55)	0.417 [0.331–0.503]	*W* = 375 0.924 (0.80)	0.302 [0.237–0.369]	*W* = 399 0.122 (0.70)
600	500	0.260 [0.198–0.321]	*W* = 367 0.089 (0.83)	26.802 [20.964–32.966]	*W* = 403 0.283 (0.63)	0.455 [0.362–0.547]	*W* = 420 0.892 (0.77)	0.301 [0.237–0.365]	*W* = 385 0.135 (0.71)

**Table A12:** Segmentation performance (mean [95% bootstrap CI]) for Myocardium, comparing the real-only baseline, GAN-based augmentation, and the corresponding diffusion-based augmentation in the $N_{\mathrm{real}}=100$ regime. Real = number of real training cases; Syn = synthetic cases added. For DSC, HD95, precision, and recall, statistical comparisons are reported versus the corresponding real-only baseline and versus diffusion using the Wilcoxon signed-rank test (two-sided, paired at the patient level), with Holm-corrected *p*-values within the three-way comparison framework. Stat. columns report the test statistic *W*, corrected *p*-value ($p_{c}$), and the rank-biserial correlation *r*. HD95 is reported in mm. Significance codes, where present, are: * p<0.05, ** p<0.01, and *** p<0.001.

Real	Syn	DSC			HD95			Precision			Recall		
		GAN	Stat. vs Real	Stat. vs DM	GAN	Stat. vs. Real	Stat. vs. DM	GAN	Stat. vs. Real	Stat. vs. DM	GAN	Stat. vs. Real	Stat. vs. DM
100	0	0.883 [0.872–0.893]	—	—	2.714 [2.366–3.106]	—	—	0.863 [0.849–0.875]	—	—	0.909 [0.896–0.920]	—	—
100	100	0.882 [0.869–0.892]	*W* = 3240 0.364 (0.55)	*W* = 2702 0.045 * (0.63)	2.716 [2.281–3.247]	*W* = 190 1.000 (0.97)	*W* = 178 1.000 (0.97)	0.871 [0.859–0.882]	*W* = 2234 <0.001 *** (0.69)	*W* = 2455 0.002 ** (0.66)	0.898 [0.883–0.909]	*W* = 1285 <0.001 *** (0.82)	*W* = 3513 0.759 (0.52)
100	200	0.883 [0.871–0.892]	*W* = 3188 0.494 (0.56)	*W* = 2860 0.132 (0.61)	2.478 [2.154–2.874]	*W* = 145.5 0.132 (0.98)	*W* = 171 0.132 (0.98)	0.872 [0.860–0.882]	*W* = 2084 <0.001 *** (0.71)	*W* = 1672 <0.001 *** (0.77)	0.898 [0.884–0.909]	*W* = 1144 <0.001 *** (0.84)	*W* = 411 <0.001 *** (0.94)
100	300	0.882 [0.872–0.891]	*W* = 3009 0.312 (0.59)	*W* = 3270 0.692 (0.55)	2.639 [2.312–3.006]	*W* = 274.5 1.000 (0.96)	*W* = 303 0.981 (0.96)	0.884 [0.872–0.895]	*W* = 675 <0.001 *** (0.91)	*W* = 1570 <0.001 *** (0.78)	0.883 [0.871–0.894]	*W* = 353 <0.001 *** (0.95)	*W* = 1020 <0.001 *** (0.86)
100	400	0.881 [0.870–0.891]	*W* = 2642 0.030 * (0.64)	*W* = 3511 0.755 (0.52)	2.554 [2.212–2.983]	*W* = 119.5 0.285 (0.98)	*W* = 194.5 0.866 (0.97)	0.874 [0.863–0.884]	*W* = 2008 <0.001 *** (0.72)	*W* = 1849 <0.001 *** (0.74)	0.892 [0.878–0.904]	*W* = 894 <0.001 *** (0.88)	*W* = 1569 <0.001 *** (0.78)
100	500	0.880 [0.868–0.889]	*W* = 2489 0.009 ** (0.66)	*W* = 3619 0.977 (0.50)	2.930 [2.503–3.416]	*W* = 330.5 1.000 (0.95)	*W* = 366 1.000 (0.95)	0.869 [0.856–0.881]	*W* = 2321 0.002 ** (0.68)	*W* = 1355 <0.001 *** (0.81)	0.894 [0.881–0.905]	*W* = 860 <0.001 *** (0.88)	*W* = 890 <0.001 *** (0.88)

**Table A13:** Segmentation performance (mean [95% bootstrap CI]) for Scar, comparing the real-only baseline, GAN-based augmentation, and the corresponding diffusion-based augmentation in the $N_{\mathrm{real}}=100$ regime. Real = number of real training cases; Syn = synthetic cases added. For DSC, HD95, precision, and recall, statistical comparisons are reported versus the corresponding real-only baseline and versus diffusion using the Wilcoxon signed-rank test (two-sided, paired at the patient level), with Holm-corrected *p*-values within the three-way comparison framework. Stat. columns report the test statistic *W*, corrected *p*-value ($p_{c}$), and the rank-biserial correlation *r*. HD95 is reported in mm. Significance codes, where present, are: * p<0.05, ** p<0.01, and *** p<0.001.

Real	Syn	DSC			HD95			Precision			Recall		
		GAN	Stat. vs. Real	Stat. vs. DM	GAN	Stat. vs. Real	Stat. vs. DM	GAN	Stat. vs. Real	Stat. vs. DM	GAN	Stat. vs. Real	Stat. vs. DM
100	0	0.173 [0.126–0.223]	—	—	29.523 [23.880–35.480]	—	—	0.456 [0.357–0.556]	—	—	0.173 [0.127–0.222]	—	—
100	100	0.181 [0.131–0.235]	*W* = 352 0.309 (0.84)	*W* = 226 <0.001 *** (0.91)	30.691 [25.018–36.678]	*W* = 375 0.841 (0.52)	*W* = 286 0.046 * (0.70)	0.437 [0.340–0.534]	*W* = 333.5 0.786 (0.77)	*W* = 356.5 0.470 (0.81)	0.193 [0.142–0.247]	*W* = 341 0.246 (0.74)	*W* = 216 <0.001 *** (0.84)
100	200	0.192 [0.145–0.242]	*W* = 190 <0.001 *** (0.92)	*W* = 235 <0.001 *** (0.92)	25.197 [20.783–29.835]	*W* = 218 0.018 * (0.73)	*W* = 292 0.018 * (0.74)	0.356 [0.277–0.437]	*W* = 167 0.009 ** (0.90)	*W* = 516 0.986 (0.80)	0.257 [0.205–0.311]	*W* = 169 <0.001 *** (0.87)	*W* = 220 <0.001 *** (0.83)
100	300	0.184 [0.137–0.234]	*W* = 287 0.025 * (0.87)	*W* = 84 <0.001 *** (0.97)	26.643 [21.632–31.982]	*W* = 238 0.110 (0.69)	*W* = 336 0.110 (0.66)	0.389 [0.302–0.477]	*W* = 292 0.738 (0.81)	*W* = 461 0.738 (0.79)	0.215 [0.166–0.267]	*W* = 264 0.012 * (0.80)	*W* = 68 <0.001 *** (0.95)
100	400	0.200 [0.148–0.254]	*W* = 157 <0.001 *** (0.93)	*W* = 161 <0.001 *** (0.94)	25.649 [20.451–31.145]	*W* = 257 0.102 (0.65)	*W* = 276 0.020 * (0.72)	0.339 [0.259–0.420]	*W* = 87 <0.001 *** (0.94)	*W* = 443 0.717 (0.81)	0.270 [0.212–0.329]	*W* = 123 <0.001 *** (0.91)	*W* = 129 <0.001 *** (0.90)
100	500	0.195 [0.140–0.250]	*W* = 329 0.126 (0.84)	*W* = 141 <0.001 *** (0.94)	28.616 [23.139–34.470]	*W* = 271 0.230 (0.61)	*W* = 266 0.066 (0.72)	0.400 [0.306–0.494]	*W* = 161 0.014 * (0.87)	*W* = 448 0.965 (0.76)	0.211 [0.157–0.269]	*W* = 288 0.041 * (0.78)	*W* = 140 <0.001 *** (0.89)

**Table A14:** Segmentation performance (mean [95% bootstrap CI]) for Myocardium, comparing the real-only baseline, GAN-based augmentation, and the corresponding diffusion-based augmentation in the $N_{\mathrm{real}}=300$ regime. Real = number of real training cases; Syn = synthetic cases added. For DSC, HD95, precision, and recall, statistical comparisons are reported versus the corresponding real-only baseline and versus diffusion using the Wilcoxon signed-rank test (two-sided, paired at the patient level), with Holm-corrected *p*-values within the three-way comparison framework. Stat. columns report the test statistic *W*, corrected *p*-value ($p_{c}$), and the rank-biserial correlation *r*. HD95 is reported in mm. Significance codes, where present, are: * p<0.05, ** p<0.01, and *** p<0.001.

Real	Syn	DSC			HD95			Precision			Recall		
		GAN	Stat. vs. Real	Stat. vs. DM	GAN	Stat. vs. Real	Stat. vs. DM	GAN	Stat. vs. Real	Stat. vs. DM	GAN	Stat. vs. Real	Stat. vs. DM
300	0	0.888 [0.878–0.897]	—	—	2.484 [2.177–2.832]	—	—	0.875 [0.862–0.887]	—	—	0.905 [0.893–0.915]	—	—
300	100	0.882 [0.871–0.891]	*W* = 1753 <0.001 *** (0.76)	*W* = 2870 0.047 * (0.60)	2.656 [2.330–3.030]	*W* = 142 0.249 (0.98)	*W* = 150.5 0.525 (0.98)	0.879 [0.867–0.891]	*W* = 2739 0.060 (0.62)	*W* = 3367 0.491 (0.54)	0.887 [0.874–0.899]	*W* = 883 <0.001 *** (0.88)	*W* = 2153 <0.001 *** (0.70)
300	200	0.880 [0.868–0.889]	*W* = 1316 <0.001 *** (0.82)	*W* = 3261 0.334 (0.55)	2.793 [2.352–3.331]	*W* = 137.5 0.249 (0.98)	*W* = 170 0.782 (0.98)	0.877 [0.865–0.888]	*W* = 3204 0.265 (0.56)	*W* = 780 <0.001 *** (0.89)	0.887 [0.873–0.898]	*W* = 704 <0.001 *** (0.90)	*W* = 457 <0.001 *** (0.94)
300	300	0.880 [0.869–0.890]	*W* = 1674 <0.001 *** (0.77)	*W* = 2843 0.039 * (0.61)	2.607 [2.258–3.036]	*W* = 153.5 0.518 (0.98)	*W* = 154 0.584 (0.98)	0.859 [0.847–0.869]	*W* = 761 <0.001 *** (0.90)	*W* = 2945 0.073 (0.59)	0.908 [0.894–0.919]	*W* = 2681 0.026 * (0.63)	*W* = 3289 0.456 (0.55)
300	400	0.878 [0.867–0.888]	*W* = 1296 <0.001 *** (0.82)	*W* = 3261 0.334 (0.55)	2.856 [2.475–3.299]	*W* = 76 0.012 * (0.99)	*W* = 161 0.024 * (0.98)	0.886 [0.875–0.897]	*W* = 1733 <0.001 *** (0.76)	*W* = 14 <0.001 *** (1.00)	0.875 [0.861–0.887]	*W* = 202 <0.001 *** (0.97)	*W* = 0 <0.001 *** (1.00)
300	500	0.877 [0.864–0.887]	*W* = 1005 <0.001 *** (0.86)	*W* = 1465 <0.001 *** (0.80)	2.786 [2.413–3.223]	*W* = 166.5 0.051 (0.98)	*W* = 137 0.051 (0.98)	0.878 [0.866–0.888]	*W* = 2813 0.064 (0.61)	*W* = 3018 0.109 (0.58)	0.881 [0.865–0.893]	*W* = 436 <0.001 *** (0.94)	*W* = 1802 <0.001 *** (0.75)

**Table A15:** Segmentation performance (mean [95% bootstrap CI]) for Scar, comparing the real-only baseline, GAN-based augmentation, and the corresponding diffusion-based augmentation in the $N_{\mathrm{real}}=300$ regime. Real = number of real training cases; Syn = synthetic cases added. For DSC, HD95, precision, and recall, statistical comparisons are reported versus the corresponding real-only baseline and versus diffusion using the Wilcoxon signed-rank test (two-sided, paired at the patient level), with Holm-corrected *p*-values within the three-way comparison framework. Stat. columns report the test statistic *W*, corrected *p*-value ($p_{c}$), and the rank-biserial correlation *r*. HD95 is reported in mm. Significance codes, where present, are: * p<0.05, ** p<0.01, and *** p<0.001.

Real	Syn	DSC			HD95			Precision			Recall		
		GAN	Stat. vs. Real	Stat. vs. DM	GAN	Stat. vs. Real	Stat. vs. DM	GAN	Stat. vs. Real	Stat. vs. DM	GAN	Stat. vs. Real	Stat. vs. DM
300	0	0.232 [0.177–0.286]	—	—	23.577 [19.208–28.363]	—	—	0.363 [0.287–0.438]	—	—	0.355 [0.285–0.426]	—	—
300	100	0.187 [0.136–0.240]	*W* = 150 <0.001 *** (0.94)	*W* = 246 0.027 * (0.89)	28.371 [22.757–34.187]	*W* = 281 0.060 (0.70)	*W* = 296 0.125 (0.66)	0.382 [0.294–0.471]	*W* = 398 0.674 (0.80)	*W* = 165 0.015 * (0.90)	0.216 [0.163–0.272]	*W* = 115 <0.001 *** (0.91)	*W* = 261 0.072 (0.80)
300	200	0.167 [0.119–0.218]	*W* = 130 <0.001 *** (0.95)	*W* = 57 <0.001 *** (0.98)	27.142 [21.654–32.992]	*W* = 222 0.022 * (0.73)	*W* = 145 0.003 ** (0.82)	0.400 [0.307–0.494]	*W* = 350 1.000 (0.82)	*W* = 240 0.279 (0.86)	0.178 [0.130–0.230]	*W* = 86 <0.001 *** (0.94)	*W* = 42 <0.001 *** (0.97)
300	300	0.164 [0.115–0.216]	*W* = 84 <0.001 *** (0.97)	*W* = 69 <0.001 *** (0.97)	30.386 [24.312–36.797]	*W* = 173 0.002 ** (0.79)	*W* = 87.5 <0.001 *** (0.89)	0.407 [0.312–0.506]	*W* = 293 0.522 (0.84)	*W* = 197 0.099 (0.89)	0.174 [0.125–0.226]	*W* = 54 <0.001 *** (0.96)	*W* = 45 <0.001 *** (0.97)
300	400	0.198 [0.145–0.252]	*W* = 137 <0.001 *** (0.95)	*W* = 163 <0.001 *** (0.93)	26.245 [20.998–31.929]	*W* = 234 0.022 * (0.75)	*W* = 166 0.003 ** (0.82)	0.437 [0.344–0.529]	*W* = 227 0.015 * (0.89)	*W* = 407 0.761 (0.79)	0.217 [0.165–0.272]	*W* = 92 <0.001 *** (0.93)	*W* = 125 <0.001 *** (0.91)
300	500	0.177 [0.129–0.228]	*W* = 130 <0.001 *** (0.96)	*W* = 166 <0.001 *** (0.93)	28.903 [23.496–34.563]	*W* = 224 0.006 ** (0.78)	*W* = 225 0.003 ** (0.77)	0.358 [0.275–0.442]	*W* = 446 0.744 (0.83)	*W* = 340 0.489 (0.83)	0.218 [0.164–0.274]	*W* = 88 <0.001 *** (0.93)	*W* = 160 <0.001 *** (0.88)

**Table A16:** Segmentation performance (mean [95% bootstrap CI]) for Myocardium, comparing the real-only baseline, GAN-based augmentation, and the corresponding diffusion-based augmentation in the $N_{\mathrm{real}}=600$ regime. Real = number of real training cases; Syn = synthetic cases added. For DSC, HD95, precision, and recall, statistical comparisons are reported versus the corresponding real-only baseline and versus diffusion using the Wilcoxon signed-rank test (two-sided, paired at the patient level), with Holm-corrected *p*-values within the three-way comparison framework. Stat. columns report the test statistic *W*, corrected *p*-value ($p_{c}$), and the rank-biserial correlation *r*. HD95 is reported in mm. Significance codes, where present, are: * p<0.05, ** p<0.01, and *** p<0.001.

Real	Syn	DSC			HD95			Precision			Recall		
		GAN	Stat. vs. Real	Stat. vs. DM	GAN	Stat. vs. Real	Stat. vs. DM	GAN	Stat. vs. Real	Stat. vs. DM	GAN	Stat. vs. Real	Stat. vs. DM
600	0	0.892 [0.883–0.900]	—	—	2.650 [2.258–3.090]	—	—	0.879 [0.867–0.890]	—	—	0.909 [0.899–0.918]	—	—
600	100	0.876 [0.863–0.887]	*W* = 641 <0.001 *** (0.91)	*W* = 2200 <0.001 *** (0.70)	2.845 [2.432–3.346]	*W* = 235.5 0.248 (0.97)	*W* = 237 0.436 (0.97)	0.871 [0.859–0.881]	*W* = 1897 <0.001 *** (0.74)	*W* = 2456 0.004 ** (0.66)	0.886 [0.870–0.899]	*W* = 715 <0.001 *** (0.90)	*W* = 3107 0.171 (0.57)
600	200	0.878 [0.866–0.888]	*W* = 691 <0.001 *** (0.91)	*W* = 1511 <0.001 *** (0.79)	2.635 [2.247–3.150]	*W* = 252.5 0.831 (0.96)	*W* = 206 0.831 (0.97)	0.875 [0.863–0.885]	*W* = 2473 0.002 ** (0.66)	*W* = 1094 <0.001 *** (0.85)	0.886 [0.871–0.898]	*W* = 500 <0.001 *** (0.93)	*W* = 3207 0.268 (0.56)
600	300	0.878 [0.864–0.888]	*W* = 782 <0.001 *** (0.89)	*W* = 2187 <0.001 *** (0.70)	2.745 [2.338–3.256]	*W* = 231.5 0.330 (0.97)	*W* = 265.5 0.834 (0.96)	0.883 [0.872–0.893]	*W* = 3271 0.347 (0.55)	*W* = 1222 <0.001 *** (0.83)	0.877 [0.861–0.890]	*W* = 109 <0.001 *** (0.98)	*W* = 214 <0.001 *** (0.97)
600	400	0.878 [0.865–0.888]	*W* = 616 <0.001 *** (0.92)	*W* = 3158 0.216 (0.56)	3.143 [2.561–3.866]	*W* = 242.5 0.045 * (0.97)	*W* = 252.5 0.248 (0.96)	0.867 [0.854–0.878]	*W* = 1345 <0.001 *** (0.81)	*W* = 2464 0.002 ** (0.66)	0.893 [0.879–0.905]	*W* = 1266 <0.001 *** (0.83)	*W* = 1530 <0.001 *** (0.79)
600	500	0.879 [0.867–0.888]	*W* = 736 <0.001 *** (0.90)	*W* = 2567 0.005 ** (0.65)	2.761 [2.337–3.297]	*W* = 252.5 0.615 (0.96)	*W* = 345.5 0.928 (0.95)	0.878 [0.867–0.888]	*W* = 2723 0.018 * (0.62)	*W* = 805 <0.001 *** (0.89)	0.884 [0.869–0.896]	*W* = 327 <0.001 *** (0.95)	*W* = 181 <0.001 *** (0.97)

**Table A17:** Segmentation performance (mean [95% bootstrap CI]) for Scar, comparing the real-only baseline, GAN-based augmentation, and the corresponding diffusion-based augmentation in the $N_{\mathrm{real}}=600$ regime. Real = number of real training cases; Syn = synthetic cases added. For DSC, HD95, precision, and recall, statistical comparisons are reported versus the corresponding real-only baseline and versus diffusion using the Wilcoxon signed-rank test (two-sided, paired at the patient level), with Holm-corrected *p*-values within the three-way comparison framework. Stat. columns report the test statistic *W*, corrected *p*-value ($p_{c}$), and the rank-biserial correlation *r*. HD95 is reported in mm. Significance codes, where present, are: * p<0.05, ** p<0.01, and *** p<0.001.

Real	Syn	DSC			HD95			Precision			Recall		
		GAN	Stat. vs. Real	Stat. vs. DM	GAN	Stat. vs. Real	Stat. vs. DM	GAN	Stat. vs. Real	Stat. vs. DM	GAN	Stat. vs. Real	Stat. vs. DM
600	0	0.295 [0.229–0.362]	—	—	24.938 [19.671–30.588]	—	—	0.469 [0.377–0.560]	—	—	0.349 [0.278–0.420]	—	—
600	100	0.162 [0.114–0.212]	*W* = 38 <0.001 *** (0.98)	*W* = 88 <0.001 *** (0.96)	31.673 [26.058–37.638]	*W* = 159 <0.001 *** (0.82)	*W* = 140 <0.001 *** (0.84)	0.488 [0.388–0.586]	*W* = 297 0.258 (0.79)	*W* = 196 0.021 * (0.89)	0.152 [0.108–0.200]	*W* = 22 <0.001 *** (0.98)	*W* = 54 <0.001 *** (0.96)
600	200	0.154 [0.105–0.206]	*W* = 6 <0.001 *** (1.00)	*W* = 27 <0.001 *** (0.99)	32.383 [26.487–38.695]	*W* = 92 <0.001 *** (0.86)	*W* = 40 <0.001 *** (0.94)	0.582 [0.467–0.694]	*W* = 176 0.046 * (0.81)	*W* = 165 0.042 * (0.85)	0.130 [0.086–0.176]	*W* = 3 <0.001 *** (1.00)	*W* = 16 <0.001 *** (0.99)
600	300	0.160 [0.111–0.211]	*W* = 49 <0.001 *** (0.97)	*W* = 68 <0.001 *** (0.97)	30.647 [24.242–37.349]	*W* = 177 0.008 ** (0.76)	*W* = 95 <0.001 *** (0.87)	0.473 [0.367–0.576]	*W* = 299 1.000 (0.75)	*W* = 237 0.393 (0.85)	0.153 [0.106–0.202]	*W* = 37 <0.001 *** (0.97)	*W* = 61 <0.001 *** (0.95)
600	400	0.145 [0.099–0.195]	*W* = 9 <0.001 *** (0.99)	*W* = 32 <0.001 *** (0.98)	30.457 [24.367–36.813]	*W* = 69 <0.001 *** (0.88)	*W* = 52 <0.001 *** (0.91)	0.515 [0.402–0.626]	*W* = 281 0.924 (0.67)	*W* = 196 0.924 (0.78)	0.125 [0.084–0.169]	*W* = 8 <0.001 *** (0.99)	*W* = 16 <0.001 *** (0.99)
600	500	0.146 [0.098–0.197]	*W* = 23 <0.001 *** (0.99)	*W* = 75 <0.001 *** (0.96)	30.520 [24.492–36.924]	*W* = 100 <0.001 *** (0.83)	*W* = 102 0.002 ** (0.82)	0.528 [0.411–0.638]	*W* = 207 0.244 (0.78)	*W* = 119 0.021 * (0.87)	0.126 [0.085–0.170]	*W* = 12 <0.001 *** (0.99)	*W* = 47 <0.001 *** (0.96)

## References

[1] Ismail T.F., Strugnell W., Coletti C., Božić-Iven M., Weingärtner S., Hammernik K., Correia T., Küstner T. (2022). Cardiac MR: From Theory to Practice. Front. Cardiovasc. Med..

[2] Aquaro G.D., De Gori C., Faggioni L., Parisella M.L., Cioni D., Lencioni R., Neri E. (2023). Diagnostic and prognostic role of late gadolinium enhancement in cardiomyopathies. Eur. Heart J. Suppl..

[3] Kazerouni A., Aghdam E.K., Heidari M., Azad R., Fayyaz M., Hacihaliloglu I., Merhof D. (2023). Diffusion models in medical imaging: A comprehensive survey. Med. Image Anal..

[4] Khosravi B., Rouzrokh P., Mickley J.P., Faghani S., Mulford K., Yang L., Larson A.N., Howe B.M., Erickson B.J., Taunton M.J. (2023). Few-shot biomedical image segmentation using diffusion models: Beyond image generation. Comput. Methods Programs Biomed..

[5] Müller-Franzes G., Niehues J.M., Khader F., Arasteh S.T., Haarburger C., Kuhl C., Wang T., Han T., Nolte T., Nebelung S. (2023). A multimodal comparison of latent denoising diffusion probabilistic models and generative adversarial networks for medical image synthesis. Sci. Rep..

[6] Bishop C.M., Bishop H. (2023). Deep Learning: Foundations and Concepts.

[7] Li S., Wu C., Feng C., Bian Z., Dai Y., Wu L.-M. (2025). Segmentation of the Left Ventricle and Its Pathologies for Acute Myocardial Infarction After Reperfusion in LGE-CMR Images. IEEE Trans. Med. Imaging.

[8] Sendra-Balcells C., Campello V.M., Martín-Isla C., Viladés D., Descalzo M.L.M., Guala A., Rodriguez-Palomares J.F., Lekadir K. (2022). Domain generalization in deep learning for contrast-enhanced imaging. Comput. Biol. Med..

[9] Jafari M., Shoeibi A., Khodatars M., Ghassemi N., Moridian P., Alizadehsani R., Khosravi A., Ling S.H., Delfan N., Zhang Y.-D. (2023). Automated diagnosis of cardiovascular diseases from cardiac magnetic resonance imaging using deep learning models: A review. Comput. Biol. Med..

[10] Kurzendorfer T., Forman C., Schmidt M., Tillmanns C., Maier A., Brost A. (2017). Fully automatic segmentation of left ventricular anatomy in 3-D LGE-MRI. Comput. Med. Imaging Graph..

[11] Mamalakis M., Garg P., Nelson T., Lee J., Wild J.M., Clayton R.H. (2021). MA-SOCRATIS: An automatic pipeline for robust segmentation of the left ventricle and scar. Comput. Med. Imaging Graph..

[12] Mamalakis M., Garg P., Nelson T., Lee J., Swift A.J., Wild J.M., Clayton R.H. (2023). Automatic development of 3D anatomical models of border zone and core scar regions in the left ventricle. Comput. Med. Imaging Graph..

[13] Mamalakis M., Garg P., Nelson T., Lee J., Swift A.J., Wild J.M., Clayton R.H. (2023). Artificial Intelligence framework with traditional computer vision and deep learning approaches for optimal automatic segmentation of left ventricle with scar. Artif. Intell. Med..

[14] Wang X., Yang S., Fang Y., Wei Y., Wang M., Zhang J., Han X. (2021). SK-UNet: An improved U-Net model with selective kernel for the segmentation of LGE cardiac MR images. IEEE Sens. J..

[15] Heidenreich J.F., Gassenmaier T., Ankenbrand M.J., Bley T.A., Wech T. (2021). Self-configuring nnU-net pipeline enables fully automatic infarct segmentation in late enhancement MRI after myocardial infarction. Eur. J. Radiol..

[16] Levchuk A.G., Bendahan D., Ryzhkov A.V., Fokin V.A., Vladimirov N., Matveev I.Y., Efimtsev A.Y., Brui E.A. Fully automatic segmentation of post-infarction fibrosis in post-contrast magnetic resonance images of heart: Preliminary study. Proceedings of the 2022 IEEE International Multi-Conference on Engineering, Computer and Information Sciences (SIBIRCON).

[17] Damit D.S.A., Hilmi A.I.N., Sulaiman S.N., Osman M.K., Karim N.K.A., Leh N.A.M. Impact of Class Labeling on Myocardium Segmentation Using Cascaded Deep Learning for Improved Myocardial Infarction Segmentation. Proceedings of the 2024 IEEE 14th International Conference on Control System, Computing and Engineering (ICCSCE).

[18] Brahim K., Arega T.W., Boucher A., Bricq S., Sakly A., Meriaudeau F. (2022). An improved 3D deep learning-based segmentation of left ventricular myocardial diseases from delayed-enhancement MRI with inclusion and classification prior information U-Net (ICPIU-Net). Sensors.

[19] Schwab M., Pamminger M., Kremser C., Obmann D., Haltmeier M., Mayr A. (2025). Error correcting 2D-3D cascaded network for myocardial infarct scar segmentation on late gadolinium enhancement cardiac magnetic resonance images. Med. Image Anal..

[20] Lin M., Jiang M., Zhao M., Ukwatta E., White J.A., Chiu B. (2022). Cascaded triplanar autoencoder M-Net for fully automatic segmentation of left ventricle myocardial scar from three-dimensional late gadolinium-enhanced MR images. IEEE J. Biomed. Health Inform..

[21] Fahmy A.S., Rowin E.J., Chan R.H., Manning W.J., Maron M.S., Nezafat R. (2021). Improved quantification of myocardium scar in late gadolinium enhancement images: Deep learning based image fusion approach. J. Magn. Reson. Imaging.

[22] Chen D., Bhopalwala H., Dewaswala N., Arunachalam S.P., Enayati M., Farahani N.Z., Pasupathy K., Lokineni S., Bos J.M., Noseworthy P.A. (2022). Deep neural network for cardiac magnetic resonance image segmentation. J. Imaging.

[23] Wang K.-N., Yang X., Miao J., Li L., Yao J., Zhou P., Xue W., Zhou G.-Q., Zhuang X., Ni D. (2022). AWSnet: An auto-weighted supervision attention network for myocardial scar and edema segmentation in multi-sequence cardiac magnetic resonance images. Med. Image Anal..

[24] Liu J., Wei A., Cao L., He X., Tang C. (2025). Contrastive Trustworthy Prototype Learning for multi-modality myocardial pathology segmentation. Appl. Soft Comput..

[25] Vesal S., Gu M., Kosti R., Maier A., Ravikumar N. (2021). Adapt everywhere: Unsupervised adaptation of point-clouds and entropy minimization for multi-modal cardiac image segmentation. IEEE Trans. Med. Imaging.

[26] Qayyum A., Razzak I., Mazher M., Lu X., Niederer S.A. (2024). Unsupervised unpaired multiple fusion adaptation aided with self-attention generative adversarial network for scar tissues segmentation framework. Inf. Fusion.

[27] Hoh T., Margolis I., Weine J., Joyce T., Manka R., Weisskopf M., Cesarovic N., Fuetterer M., Kozerke S. (2024). Impact of late gadolinium enhancement image acquisition resolution on neural network based automatic scar segmentation. J. Cardiovasc. Magn. Reson..

[28] Wang H., Huang H., Wu J., Li N., Gu K., Wu X. (2024). Semi-supervised segmentation of cardiac chambers from LGE-CMR using feature consistency awareness. BMC Cardiovasc. Disord..

[29] Lustermans D.R.P.R.M., Amirrajab S., Veta M., Breeuwer M., Scannell C.M. (2022). Optimized automated cardiac MR scar quantification with GAN-based data augmentation. Comput. Methods Programs Biomed..

[30] Konz N., Chen Y., Dong H., Mazurowski M.A. Anatomically-Controllable Medical Image Generation with Segmentation-Guided Diffusion Models. Proceedings of the International Conference on Medical Image Computing and Computer-Assisted Intervention (MICCAI).

[31] Croitoru F.-A., Hondru V., Ionescu R.T., Shah M. (2023). Diffusion models in vision: A survey. IEEE Trans. Pattern Anal. Mach. Intell..

[32] Kebaili A., Lapuyade-Lahorgue J., Ruan S. (2023). Deep learning approaches for data augmentation in medical imaging: A review. J. Imaging.

[33] Kebaili A., Lapuyade-Lahorgue J., Vera P., Ruan S. (2024). 3D MRI Synthesis with Slice-Based Latent Diffusion Models: Improving Tumor Segmentation Tasks in Data-Scarce Regimes. arXiv.

[34] Khader F., Müller-Franzes G., Tayebi Arasteh S., Han T., Haarburger C., Schulze-Hagen M., Schad P., Engelhardt S., Baeßler B., Foersch S. (2023). Denoising diffusion probabilistic models for 3D medical image generation. Sci. Rep..

[35] Han K., Xiong Y., You C., Khosravi P., Sun S., Yan X., Duncan J.S., Xie X. MedGen3D: A Deep Generative Framework for Paired 3D Image and Mask Generation. Proceedings of the International Conference on Medical Image Computing and Computer-Assisted Intervention (MICCAI).

[36] Zhang J., Luo G., Zhang Z., Zhu Y. Data Augmentation in Class-Conditional Diffusion Model for Semi-Supervised Medical Image Segmentation. Proceedings of the 2024 International Joint Conference on Neural Networks (IJCNN).

[37] Zhou X., Zhang B., Zhang T., Zhang P., Bao J., Chen D., Zhang Z., Wen F. CoCosNet v2: Full-Resolution Correspondence Learning for Image Translation. Proceedings of the IEEE/CVF Conference on Computer Vision and Pattern Recognition (CVPR).

[38] Isensee F., Jaeger P.F., Kohl S.A.A., Petersen J., Maier-Hein K.H. (2021). nnU-Net: A self-configuring method for deep learning-based biomedical image segmentation. Nat. Methods.

[39] Ho J., Jain A., Abbeel P. (2020). Denoising diffusion probabilistic models. Advances in Neural Information Processing Systems (NeurIPS).

[40] Nichol A.Q., Dhariwal P. Improved denoising diffusion probabilistic models. Proceedings of the International Conference on Machine Learning (ICML).

[41] Dorjsembe Z., Pao H.-K., Odonchimed S., Xiao F. (2024). Conditional diffusion models for semantic 3D brain MRI synthesis. IEEE J. Biomed. Health Inform..

[42] Seitzer M. Pytorch-fid: FID Score for PyTorch. GitHub Repository 2020 (Version 0.3.0). https://github.com/mseitzer/pytorch-fid.

[43] Gudbjartsson H., Patz S. (1995). The Rician distribution of noisy MRI data. Magn. Reson. Med..

[44] Abdulkareem M., Kenawy A.A., Rauseo E., Lee A.M., Sojoudi A., Amir-Khalili A., Lekadir K., Young A.A., Barnes M.R., Barckow P. (2022). Predicting post-contrast information from contrast agent free cardiac MRI using machine learning: Challenges and methods. Front. Cardiovasc. Med..

[45] Donoho D.L., Johnstone I.M. (1994). Ideal spatial adaptation by wavelet shrinkage. Biometrika.

[46] Coupé P., Manjón J.V., Gedamu E., Arnold D., Robles M., Collins D.L. (2010). Robust Rician noise estimation for MR images. Med. Image Anal..

